# Monitoring plant moisture content and optimizing irrigation prescriptions based on UAV multimodal data

**DOI:** 10.3389/fpls.2026.1827521

**Published:** 2026-05-15

**Authors:** Wanna Fu, Xinyue Hou, Dawei Wang, Zhen Chen, Qian Cheng, Yafeng Li, Fuyi Duan

**Affiliations:** 1 Heilongjiang Provincial Hydraulic Research Institute, Harbin, China; 2 Institute of Farmland Irrigation, Chinese Academy of Agricultural Sciences, Xinxiang, China

**Keywords:** modified evapotranspiration, plant moisture content, precision irrigation, unmanned aerial vehicle, water use efficiency

## Abstract

**Introduction:**

With the continuous advancement of smart agriculture, multi-modal remote sensing based on unmanned aerial vehicles (UAVs) offers new technical approaches for monitoring and managing crop moisture in fields. However, significant challenges remain in developing high-precision field-scale crop Plant Moisture Content (PMC) prediction models and translating them into actionable irrigation strategies.

**Methods:**

This study focuses on winter wheat, employing field experiments with PMC and water use efficiency (WUE) as indicators of crop water status. Vegetation indices (VIs) derived from UAV data were used to construct a leaf area index (LAI) inversion model. Crop Height was extracted from oblique photogrammetry point cloud data. By combining the Penman-Monteith equation with dual crop coefficients, an improved evapotranspiration (ET) model was developed, utilizing multispectral data from UAVs, thermal infrared data, point cloud-derived plant height, and LAI inversion results. Further utilizing VIs, temperature indices (TIs), and machine learning algorithms (Random Forest Regression (RFR), Back Propagation Neural Network (BPNN), Partial Least Squares Regression (PLSR), and Support Vector Regression (SVR), we established PMC prediction models for winter wheat at different growth stages. These models, integrated with WUE, form the basis for an irrigation scheduling optimization framework at the field scale.

**Results:**

Results indicate that VIs, the difference between canopy temperature and air temperature (ΔT), Crop Water Stress Index (CWSI), and ET exhibit varying correlations with PMC during three critical growth stages of winter wheat, with ET showing the highest correlation during the jointing and heading stages (absolute correlation coefficient |r| ≥ 0.639). Compared to PMC prediction models constructed with different combinations of VIs, ET, VIs+ET, and VIs+TIs, the model employing the RFR algorithm with multimodal inputs (VIS+TIs+ET) demonstrated the best performance. The model’s predictive accuracy gradually improved across all growth stages, peaking during the grain-filling stage, with the coefficient of determination(R^2^) of 0.900 and a normalized root mean square error (nRMSE) of 2.688%. Optimal WUE varied across growth stages under different irrigation treatments. The highest values were achieved at the jointing stage under treatment W3 (PMC = 81.8%), and at the heading and grain-filling stages under treatment W1 (PMC = 76.8% and 64.0%, respectively).

**Discussion:**

The study suggests that stage-specific irrigation scheduling based on PMC thresholds can improve overall water use efficiency. This study shows that integrating multi-modal UAV data with machine learning and an improved ET model enables high-precision PMC monitoring, supporting data-driven irrigation scheduling in precision agriculture.

## 1 Introduction

Climate change and the growing scarcity of global water resources pose unprecedented challenges to agriculture. Improving water use efficiency (WUE) is crucial in arid and water-scarce regions for enhancing agricultural productivity (Nhamo et al., 2019). Water deficit affects crops by influencing their physiological and biochemical processes as well as their morphology. The water status of crops not only impacts their normal growth and development but also directly determines agricultural yield and quality. Compared with soil moisture, crop water status is more closely related to physiological and biochemical functions (such as photosynthesis, transpiration, and is directly correlated with biomass and yield (Zhou et al., 2022). Numerous studies have identified leaf water content as a key indicator of crop water status. Therefore, timely acquisition and rapid diagnosis of winter wheat plant water status, coupled with precise monitoring of plant moisture content (PMC), enable the formulation of appropriate irrigation management measures to enhance WUE. This is crucial for ensuring high-quality and high-yield winter wheat production, and holds significant importance for optimizing irrigation management, improving WUE, and safeguarding food security (Ali et al., 2025).

Traditional irrigation decisions often rely on soil moisture measurements or experience-based judgments, which are time-consuming, labor-intensive, and inefficient. They struggle to accurately reflect the actual moisture status of crops and exhibit significant spatial variability. In recent years, unmanned aerial vehicle (UAV)-based remote sensing has emerged as a powerful tool for monitoring the water status of vegetation and detecting physiological changes in crops under water stress (Nowack et al., 2024). UAVs equipped with visible and multispectral sensors have revolutionized the acquisition of crop spectral information, enabling the use of advanced techniques to estimate key physiological and structural parameters such as Leaf Area Index (LAI) (Wu et al., 2023)、and PMC (Shu et al., 2022). Infrared imaging technology for unmanned aerial vehicles can detect thermal radiation emitted from plant surfaces, facilitating analysis of canopy temperature and its correlation with crop water deficit. Based on these measurements, the Temperature Index (TIs) (Carrasco-Benavides et al., 2022) has been developed and widely applied to assess crop water status. Early studies primarily established spectral prediction models for PMC by constructing VIs based on sensitive spectral bands, achieving certain results. Sun et al. (2024) employed the sequential projection algorithm (SPA) to select the sensitive bands for Leaf Water Content (LWC). Our results indicate that the irrigation quotas derived from hyperspectral inversion-estimated soil moisture content (SMC) exhibit high consistency with those based on measured SMC. The irrigation quotas derived from hyperspectral inversion-estimated SMC demonstrate high consistency with those based on measured SMC. Li et al. (2023) proposed transforming and processing hyperspectral data using the fractional differential and continuous wavelet transforms. They employed differential spectra, wavelet coefficients, and hybrid variables (combining differential spectra and wavelet coefficients) as model inputs. Gaussian process regression (GPR), classification and regression decision trees (CART), and artificial neural networks (ANN) were utilized to estimate LWC at different growth stages of wheat. Results indicate that fractional differentiation and continuous wavelet transform effectively enhance spectral sensitivity to LWC, improving both the predictive capability and stability of wheat LWC estimation. Although vegetation index-based PMC inversion methods have reached a high level of maturity in practical applications, the crop canopy spectra obtained during this process are susceptible to interference from soil background and environmental noise, resulting in data redundancy (Wu et al., 2024). Especially during the early stages of water stress, crops may regulate leaf temperature and moisture status through stomatal control. Relying solely on vegetation indices (VIs) at this time can easily lead to misjudgment. Furthermore, these models often overlook the influence of crop growth environments and intrinsic physiological characteristics on PMC, which also constitutes a significant factor limiting the models’ generalization capabilities. The water status within crops depends on both absorption and transpiration; reduced absorption or excessive transpiration can both lead to water deficit (Martinetti et al., 2024). Therefore, incorporating ET—which characterizes the water-energy coupling process—into the modeling process is crucial for overcoming the limitations of existing PMC monitoring models that rely solely on VIs. This approach enables multimodal fusion of multi-source data on crop PMC, thereby establishing precise irrigation decisions and WUE for winter wheat.

As a vital component of the global water cycle and surface energy balance, evapotranspiration (ET) significantly influences crop growth and development as well as yield formation, serving as the basis for determining optimal irrigation regimes (Zhu et al., 2025). The Food and Agriculture Organization of the United Nations (FAO) utilizes the dual crop coefficient method to estimate crop water requirements, a technique that is widely recognized as the analytical foundation for irrigation system management and real-time irrigation scheduling. This method accurately calculates crop water requirements by considering factors such as crop growth stages, canopy coverage, climatic conditions, and soil properties. While the dual crop coefficient method is both simple and highly practical, it does not address the underlying mechanisms of crop water demand. Furthermore, it lacks an explanation of how irrigation management practices should be adjusted to account for the dynamic changes in crop water requirements throughout the various stages of crop growth. Shrestha and Shukla (2014) employed the dual crop coefficient method to investigate the transpiration of mulched vines, enabling separate estimation of crop transpiration and soil evaporation. They achieved satisfactory simulation results and found that using the crop coefficient recommended by FAO-56 tends to overestimate soil evaporation in farmland. Ding et al. (2013) modified the dual crop coefficient method using corn under mulching conditions as the study subject. They found that the modified dual crop coefficient method simulated corn ET more effectively, indicating that the crop coefficients recommended by FAO-56 require adjustment based on varying climatic conditions in certain regions. Differences in crop growth environments and vigor significantly influence crop coefficients, thus often necessitating the use of modified crop coefficients to estimate field ET.

The advancement of remote sensing technology has opened new avenues for capturing the spatiotemporal distribution of ET. VIs extracted via drone-based remote sensing enable rapid assessment of crop growth conditions and provide spatial distribution information of ET at the field scale. Compared to traditional ground-based observations and satellite remote sensing, this approach offers higher resolution, more flexible operational cycles, and lower costs (Chang et al., 2025). Zhang et al. (2023) assessed maize evapotranspiration (ET) using high-resolution UAV imagery and the FAO-56 dual crop coefficient method. The crop water stress index (CWSI), based on canopy temperature and degree days above the canopy threshold (DACT), was employed to determine the stress coefficient (K_s_). Three normalized difference vegetation indices (NDVI), the soil-adjusted vegetation index (SAVI), and the enhanced vegetation index (EVI) were used to estimate the basic crop coefficient (K_cb_). Subsequently, these two forms of stress coefficients, combined with the three vegetation indices, were used to assess the estimation of daily crop evapotranspiration (ET_c_) under different irrigation treatments over two years in the southwestern region of Inner Mongolia. The results indicate that the combination of NDVI and CWSI yields the most accurate estimation of maize ET_c_. Shao et al. (2021) combined unmanned aerial vehicle (UAV) multispectral remote sensing technology with the random forest algorithm to generate high-resolution spatial distribution maps of the corn crop coefficient (K_c_) under different irrigation conditions. This method is easy to implement but lacks universality and accuracy. The primary reason is that the estimation model parameters are based solely on spectral data, failing to adequately account for the influence of crop parameters and meteorological parameters on ET. Secondly, the dual crop coefficient method for calculating ET is established under the underlying mechanisms of ET, whereas remote sensing empirical models lack such mechanistic support (Disasa et al., 2025).

Additionally, previous studies often obtained crop parameters for the dual-crop coefficient method through manual collection or satellite remote sensing inversion. Manual collection is time-consuming and labor-intensive, destructive to farmland crops, and yields only point-source data without direct access to area-source data. Satellite remote sensing, meanwhile, suffers from insufficient spatio-temporal resolution. Therefore, this study improves the dual-crop coefficient method by integrating UAV remote sensing to estimate crop parameters—specifically plant height and leaf area index—without altering the original computational framework. This combined approach enables rapid and accurate estimation of crop ET in large-scale fields.

Since VIs tend to saturate during the mid-to-late stages of crop growth, integrating parameters reflecting canopy structure becomes crucial for enhancing the accuracy of ET estimation. As a key indicator measuring crop canopy structure and coverage, LAI not only quantifies the amount of vegetation per unit area but also directly influences crop light absorption, gas exchange, and water regulation (Paul et al., 2021). Multiple studies indicate that LAI is an indispensable input variable in regional-scale ET estimation, with its importance being particularly pronounced when considering planting density and growth stages. Under remote sensing conditions, LAI can be obtained through multispectral inversion or acquired using optical instruments or devices such as lidar (LiDAR). Liu et al. (2024) developed a method for generating high-resolution temporal LAI maps for precision agriculture by estimating crop leaf area index using Sentinel-2 imagery and a coupled PROSAIL-Transformer model. Additionally, crop height serves as a crucial indicator reflecting crop growth status. Plant height is one of the key physiological parameters of crops and an indicator reflecting crop growth conditions (Roitsch et al., 2019). Traditional methods of measuring plant height typically rely on ground-based surveys, which are time-consuming, labor-intensive, and prone to causing crop damage, while yielding only discrete point-source data. Recent advancements in UAV-based 3D point cloud reconstruction, leveraging oblique photography and Structure from Motion (SfM) algorithms, facilitate the high-precision extraction of crop height at the field scale. This technological development offers a critical structural parameter that improves evapotranspiration (ET) estimation, particularly in overcoming vegetation index saturation issues during the mid-to-late growth stages. In summary, we constructed an improved ET model by integrating UAV multispectral data, thermal infrared data, point cloud-derived plant height, and LAI inversion results with the Penman-Monteith equation and dual crop coefficients. By further integrating key parameters such as multispectral VIs and temperature indices (TIs), and employing Random Forest Regression (RFR), Back Propagation Neural Network (BPNN), Partial Least Squares Regression (PLSR), and Support Vector Regression (SVR) to establish a prediction model for winter wheat PMC under different irrigation treatments and growth stages. Furthermore, by incorporating WUE, we established a field-scale dynamic irrigation scheduling framework that enables multi-temporal and controllable water management for winter wheat. The specific objectives of this study were threefold: (1) to develop a robust PMC prediction model for winter wheat by integrating multi-source UAV data and machine learning; (2) to quantify the contribution of TIs and ET in improving PMC estimation accuracy; and (3) to establish a dynamic, field-scale irrigation scheduling framework optimized for water use efficiency.

## 2 Materials and methods

### 2.1 Study area and experimental design

**Figure 1:**
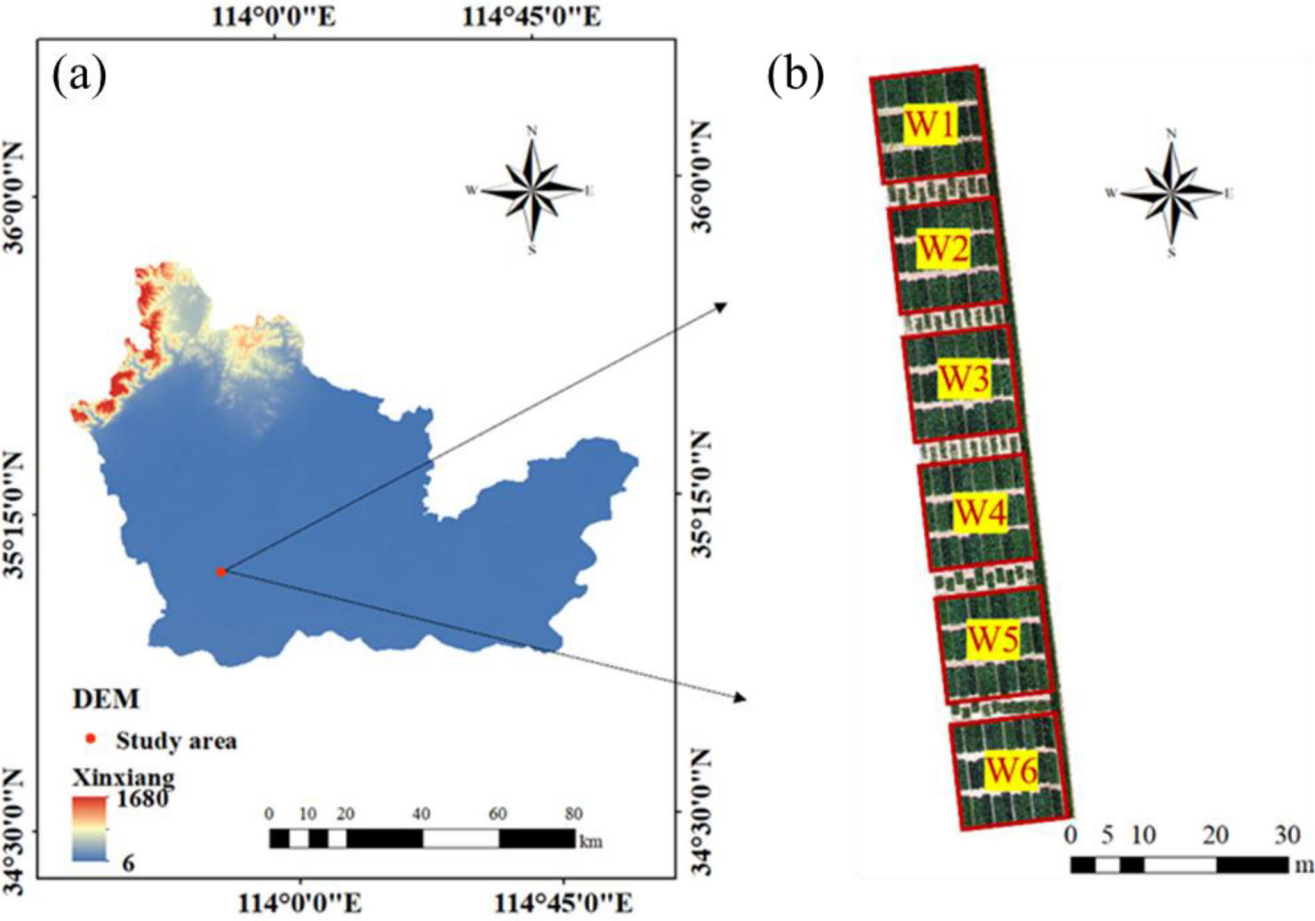
Location and experimental design of the study area. **(A)** Boundary of Xinxiang City; **(B)** Winter wheat trials in the study area.

**Figure 2:**
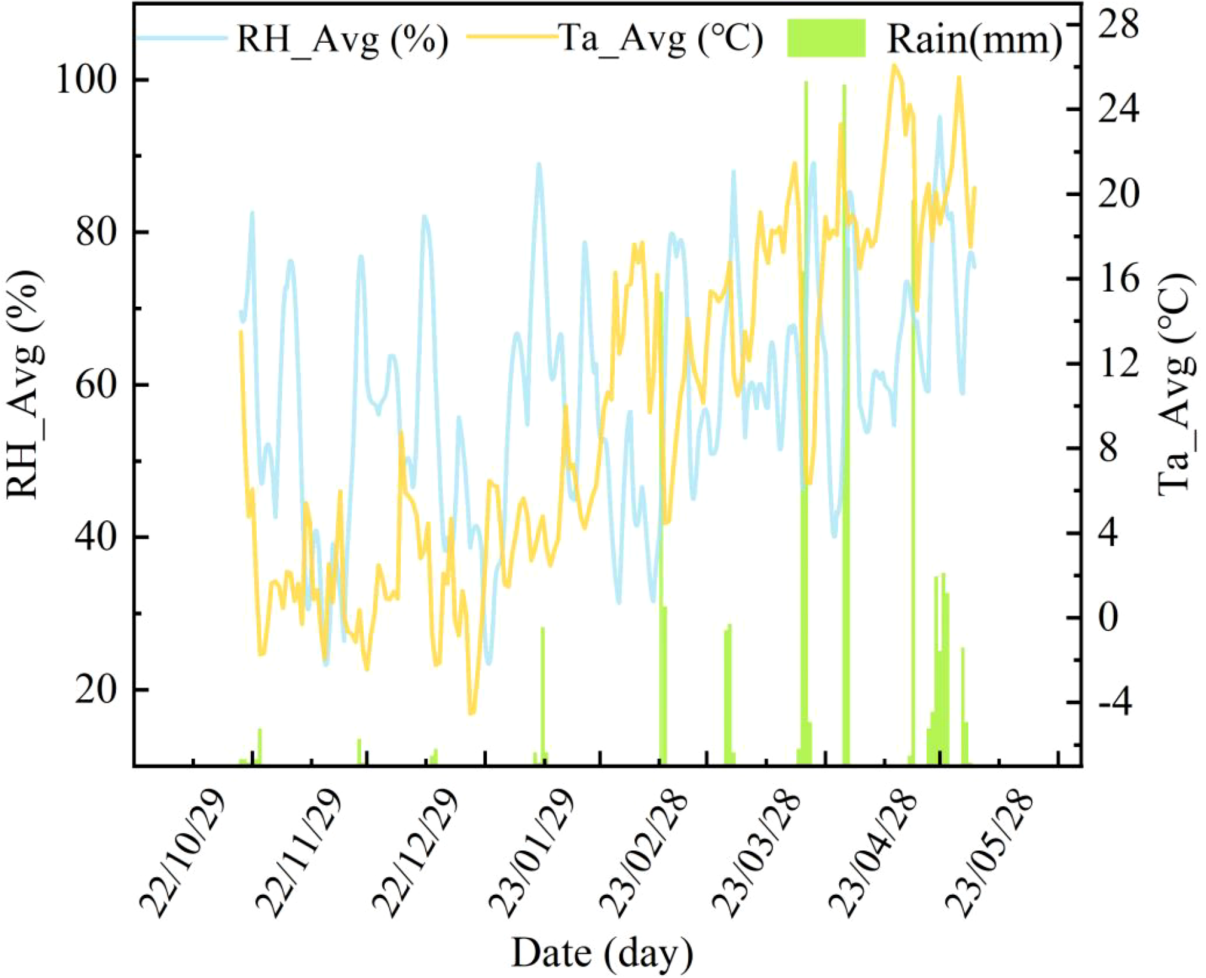
Daily meteorological data during the growth period of winter wheat in 2022–2023.

Field trials were conducted in 2023 at the Xinxiang Comprehensive Experimental Base of the Chinese Academy of Agricultural Sciences, located in Henan Province, China (113°45′42″E, 35°08′05″N). The region has a temperate monsoon climate, with an average annual temperature of 14 °C and annual precipitation of approximately 573.4 mm, providing favorable conditions for winter wheat cultivation. The local cropping system follows a year-round corn–wheat rotation on sandy loam soil. A split-plot experimental design was employed, with six irrigation treatments: W1 (282 mm), W2 (254 mm), W3 (198 mm), W4 (141 mm), W5 (85 mm), and W6 (0 mm). Each treatment consisted of 30 plots, totaling 180 plots. Each plot measured 1.4 m × 4 m, with a planting row spacing of 15 cm, and the basic seedling density was approximately 150,000 plants per mu (**Figure 1**). Nitrogen fertilizer was administered to wheat during the jointing and heading stage, with a 2:1 ratio for fertilizer distribution. All meteorological data were obtained from an *in situ* automatic weather station. Temperature and humidity variations during the experiment are presented in **Figure 2**. Routine irrigation was applied using large-scale traveling sprinklers, and other field management practices adhered to the local conditions of winter wheat production.

### 2.2 Field data collection

This study conducted experiments during the jointing, heading, and grain filling stages. To assess wheat growth and water status under varying treatment conditions, all ground sampling was scheduled 5–7 days after irrigation to fully capture differences in plant water stress. Ground measurements were performed on the same day as UAV flights. At each sampling site, six representative wheat plants of uniform growth were selected. A millimeter-precision ruler was used to measure the vertical distance from the soil surface to the tallest point of each plant, and the average height of these six plants was recorded as the representative canopy height for the plot. The maximum length and width of the uppermost intact leaf were measured using a tape measure to ensure consistency and accuracy. Leaf area per leaf was then calculated with empirical formulas, summed to obtain leaf area per plant, and further processed to determine the LAI. Simultaneously, the entire aboveground portion of each plant was collected for the determination of fresh and dry weights. Fresh weight was measured immediately, after which the samples were placed in paper bags and dried at 105 °C for 30 minutes. The oven temperature was then reduced to 75 °C, and samples were dried to constant weight, with the final dry weight recorded. PMC was calculated based on fresh and dry weight. **Table 1** presents the statistical results for LAI and crop height parameters of wheat harvested under different irrigation treatments across three growth stages.

**Table 1:** Statistics of LAI and crop height of wheat at different growth stages.

Growth stages	Sample size	Leaf area index				Crop height(m)			
		Min	Max	Mean	Standard deviation	Min	Max	Mean	Standard deviation
Jointing stage	180	3.10	3.59	3.41	0.09	0.38	0.61	0.5	0.04
Heading stage	180	5.09	5.34	5.27	0.05	0.63	0.91	0.76	0.05
Grain filling stage	180	4.78	5.15	4.94	0.07	0.62	0.96	0.79	0.05

### 2.3 Acquisition and preprocessing of UAV remote sensing data

**Figure 3:**
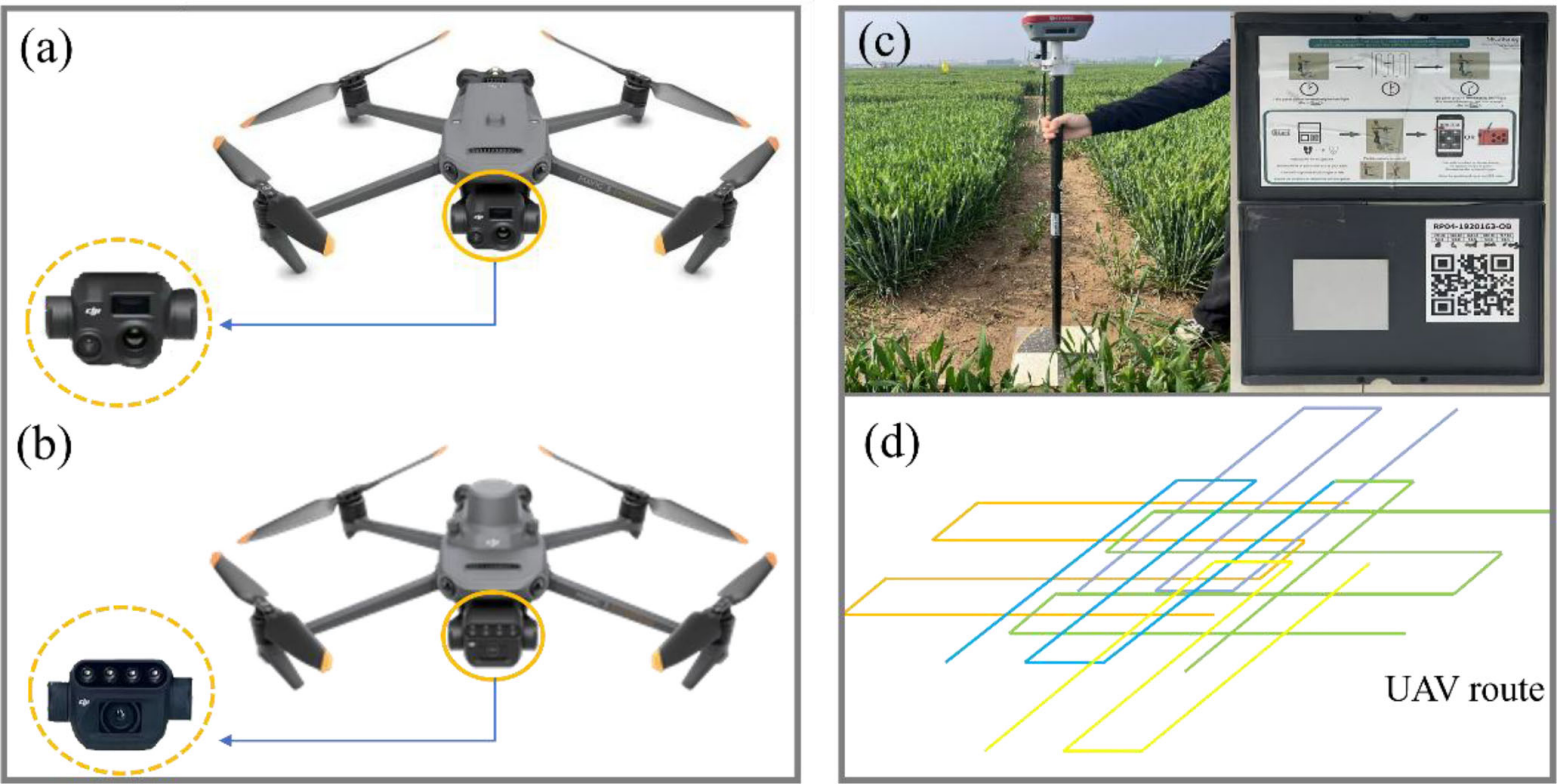
UAV remote sensing platform with radiometric calibration board and ground control points. **(A)** DJI Mavic 3T; **(B)** DJI Mavic 3M; **(C)** RTK and ground control points; **(D)** radiometric calibration board.

This study employed two unmanned aerial vehicle (UAV) platforms—the DJI Mavic 3M and Mavic 3T (DJI Innovation Technology Co., Ltd., Shenzhen, China)—for remote sensing data acquisition. To minimize the impact of solar zenith angle variations on image quality, all UAV flights were scheduled between 11:30 AM and 1:00 PM on clear days and maintained a consistent altitude of 30 m to capture high-quality imagery. The Mavic 3M drone is equipped with two sensors: an RGB sensor (20-megapixel effective resolution, 24mm equivalent focal length) and a multispectral sensor (5-megapixel effective resolution, 25mm equivalent focal length) featuring four bands: Green (G, 560 ± 16 nm), Red (R, 650 ± 16 nm), Red Edge (RE, 730 ± 16 nm), and Near-Infrared (NIR, 860 ± 26 nm). Using DJI Pilot 2 (Shenzhen DJI Innovation Technology Co., Ltd., Shenzhen, China) to plan flight routes, the camera was set to time-lapse mode. Throughout the flight, the camera remained perpendicular to the ground at a relative altitude of 30 meters. Both forward and sideways overlap rates were set to 80% to ensure sufficient image overlap for subsequent processing requirements. Before executing the UAV flight mission, a micasense calibrated reflectance panel was captured to facilitate subsequent radiometric correction of the imagery. For the acquisition of oblique photogrammetry data, the DJI Mavic 3T drone was employed. Its RGB sensor features 48 million effective pixels with an equivalent focal length of 24 millimeters. The acquisition process employed a five-directional oblique photography mode (vertical downward, forward tilt, backward tilt, left tilt, and right tilt) with a 45° tilt angle. This enabled the generation of point cloud data for winter wheat through 3D reconstruction. We processed the thermal infrared images using the “Thermal Camera” module in Pix4Dmapper software. This module automatically performs radiometric calibration and temperature extraction during the image stitching process, eliminating the need for additional manual calibration steps. Additionally, bare ground imagery was captured immediately after wheat sowing to determine bare ground elevation. To enhance the geolocation accuracy of the imagery data, 15 ground control points (GCPs) were uniformly distributed across the experimental area. These GCPs were utilized during image post-processing to improve positioning precision (**Figure 3**).

Preprocessing of drone imagery was performed using DJI Terra software (SZ DJI Technology Co., Shenzhen, China). This involved stitching RGB, MS, and TIR images captured by the drone to generate orthophotos. Key steps included ground control point (GCP) establishment, georeferencing, image registration, dense point cloud generation, digital surface model (DSM) construction, and radiometric calibration. Using the DJI Terra software (SZ DJI Technology Co., Shenzhen, China) for three-dimensional reconstruction of images obtained through oblique photography, the reconstructed point cloud data were imported into LiDAR360 (V. 5.2, Green Valley, Co. Ltd., Beijing, China) for preprocessing. This preprocessing encompassed essential tasks, including clipping, denoising, filtering, and normalization of the point cloud data, to enhance data quality and establish a foundation for subsequent analysis.

### 2.4 Vegetation indices extraction

UAV imagery from different growth stages was georeferenced using ArcGIS 10.6 (Environmental Systems Research Institute, Inc., Redlands, CA, USA). Vegetation indices (VIs) were extracted by delineating polygon vectors. VIs are derived by combining data from different spectral bands to emphasize vegetation characteristics and distinguish between vegetation and soil. They effectively mitigate the influence of factors such as land cover types and atmospheric conditions on vegetation reflectance, enabling precise monitoring of subtle changes in crops (Yan et al., 2025). Building upon previous research, this study identified and calculated multiple VIs. The corresponding formulas are listed in **Table 2**.

**Table 2:** Vegetation indices used in this study.

Vegetation indices	Formula	Reference
Normalized difference vegetation index (NDVI)	(NIR−R)/(NIR+R)	(Alizadeh et al., 2026)
Kernel normalized difference vegetation index (kNDVI)	$\tanh\left({\left(\frac{\text{NIR}-\text{R}}{2\sigma }\right)}^{2}\right)$	(Xin et al., 2026)
Renormalized difference vegetation index (RDVI)	$(\text{NIR}-\text{R})/\sqrt{\left(\text{NIR}+\text{R}\right)}$	(Ouma and Tateishi, 2011)
Modified nonlinear vegetation index (MNLI)	$1.5\left({\text{NIR}}^{2}-\text{R}\right)/\left({\text{NIR}}^{2}+\text{R}+0.5\right)$	(Feng et al., 2019)
Optimization of soil regulatory vegetation index (OSAVI)	1.16∗[(NIR−R)/(NIR+R+0.16)]	(Qiao et al., 2022)
Nonlinear vegetation index (NLI)	$\left({\text{NIR}}^{2}-\text{R})/({\text{NIR}}^{2}+\text{R}\right)$	(Huang et al., 2023)
Optimal vegetation index (Vlopt)	$1.45*({\text{NIR}}^{2}+1)/\left(\text{R}+0.45\right)$	(Chen et al., 2023)
Green chlorophyll index (CI_G_)	(NIR/G−1)	(Taniguchi et al., 2025)
vegetation supply water index (VSWI)	Ts/NDVI	(Chen et al., 2020)
Chlorophyil index rededge (Clrededge)	NIR/RE−1	(Shan et al., 2026)
Modified RETVI (MRETVI)	1.2∗[1.2∗(NIR−G)−2.5∗(RE−G)	(Dong et al., 2022)
Chlorophyll Index Red Edge (CI_RE_)	(NIR/RE−1)	(Cheng et al., 2025)
Normalized green index (NGI)	G/(NIR+G+RE)	(Zhang et al., 2025)
Red edge ratio vegetation index (RERVI)	NIR/RE	(Barzin et al., 2022)
Normalized Difference Water Index (NDWI)	(G−NIR)/(G+NIR)	(Chai et al., 2021)
Difference vegetation index (DVI)	NIR−R	(Shi et al., 2026)
Soil-Adjusted vegetation index (SAVI)	(NIR−R)(1+l)/(NIR+R+l)(l=0.5)	(Silva et al., 2025)
Modified soil-adjusted vegetation index (MSAVI2)	$(2*\text{NIR}+1-\text{SQRT(}{\left(2*\text{NIR}+1\right)}^{2}-8*(\text{NIR}-\text{R})))/2$	(Teerawong et al., 2014)

R, red band reflectivity, G, green band reflectivity, B, blue band reflectivity, NIR, near infrared band reflectivity, EDGE, red edge band reflectivity. α=0.12, σ=0.5(NIR+RED).

### 2.5 Indicator calculation

#### 2.5.1 Calculation of crop moisture content indicators

PMC is defined as the ratio of the difference between above-ground fresh weight (PFW) and plant dry weight (PDW) to PFW (Kanneh et al., 2026). The calculation formula is shown in Equation 1:

(1) $$PMC_{i}(\%)=\frac{PFW_{i}-PDW_{i}}{PFW_{i}}*100$$

WUE models form the foundation of many crop models. WUE represents the dry biomass obtained per unit of water consumed by crops. Biomass is the product of the time integral of actual transpiration T_c,adj_ and WUE, The calculation formula is shown in Equation 2:

(2) $$m_{Bio}=WUE\int_{t_{0}}^{t}T_{c,adj}dt$$

In the formula, m_Bio_ represents the above-ground stem biomass accumulation during the time period, g/m^2^;T_0_ is the emergence date;t is the biomass acquisition date;WUE-- crop water use efficiency, kg/m^3^.

#### 2.5.2 Calculation of temperature index

Multispectral and thermal infrared orthomosaics were imported into ArcGIS for further processing. Based on canopy temperature data, two thermal indices (TIs) were calculated: the difference between canopy temperature and air temperature (ΔT, °C) and CWSI. The calculation formula is shown in Equation 3:

(3) $$\Delta T=T_{c}-T_{a}$$

In the formula, Tc represents canopy temperature, and Ta represents air temperature.

The method for calculating CWSI dates back to 1990, when research indicated that changes in leaf temperature were directly correlated with crop water stress (Fuchs, 1990). The calculation formula is shown in Equation 4:

(4) $$CWSI=\frac{T_{c}-T_{wet}}{T_{dry}-T_{wet}}$$

**Figure 4:**
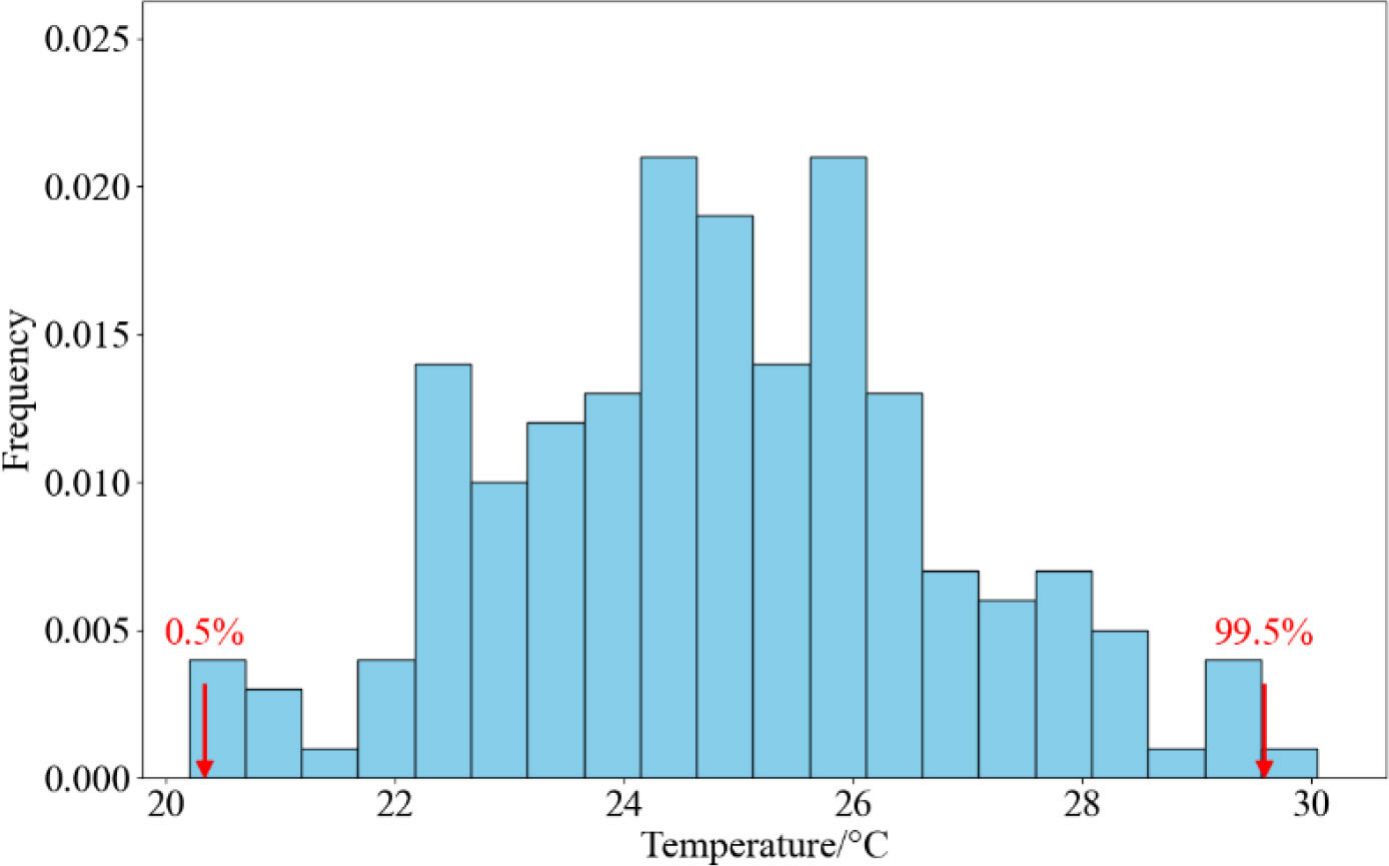
Frequency histogram of canopy temperature.

In the formula, *T*_c_ represents the average canopy temperature (°C) for each square, while *T_wet_* and *T_dry_* correspond to the canopy temperatures at maximum transpiration rate and complete absence of transpiration, respectively. In ArcGIS, a frequency histogram of canopy temperatures was calculated for each grid cell. The temperature distribution of the wheat canopy roughly followed a Gaussian distribution. In this study, *T_wet_* and *T_dry_* were taken from the 0.5% and 99.5% percentiles of the mean wheat canopy temperature histogram, respectively (as shown in **Figure 4**).

### 2.6 Leaf area index retrieval

LAI is closely related to canopy structure, leaf number, and size, exerting a strong influence on crop transpiration. When crops experience water stress, leaf growth is affected (i.e., leaf curling). Changes in LAI also impact canopy spectral information. This study employs RFR as the inversion model, primarily due to its demonstrated high accuracy and robustness in previous research for LAI inversion tasks (Yang et al., 2023). The following VIs (RDVI, OSAVI, Vlopt, MNLI, SAVI, NDVI), widely used in crop growth monitoring, were selected as inputs for the RFR-based LAI inversion model. Subsequently, the model was implemented in a Python 3.9.12 environment using machine learning regression functions from the Scikit-Learn (1.0.2) package. Model parameters were configured as follows: These parameters were used to develop an LAI inversion model, which was validated with field measurement data.

### 2.7 Crop height extraction

**Figure 5:**
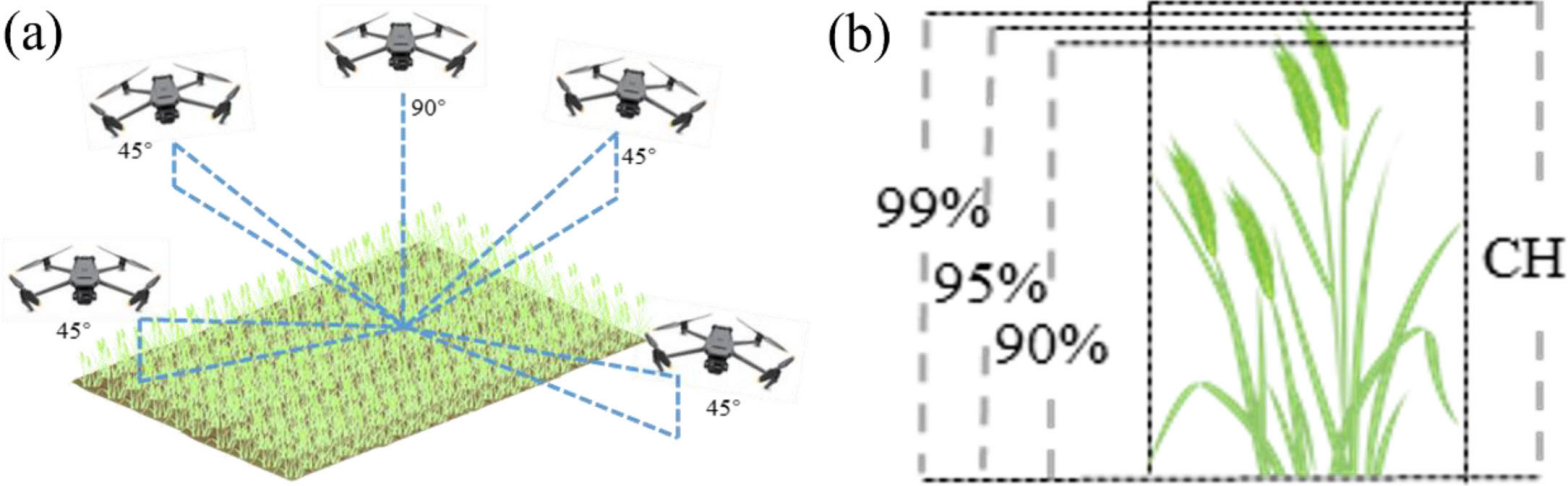
Location of drone-captured photographs and AIH method. CH, crop height. **(A)** Schematic diagram of five-directional oblique photography; **(B)** Schematic diagram of cumulative height percentiles.

Wheat plant height generally follows a pattern of rapid growth followed by stabilization. From the jointing stage to the heading stage, wheat exhibits relatively rapid developmental growth. Upon entering the grain filling stage, the trend of wheat plant height changes slows down, and plant height decreases slightly during the late phenological period. By sorting point cloud data in ascending order based on height values, an Accumulated Height Histogram (AIH) is constructed. AIH is a representation method based on the height distribution of point clouds, which can be used to estimate the vertical structural characteristics of ground features. In the AIH method, by selecting a lower AIH value as the lower boundary and a higher AIH value as the upper boundary of the vegetation canopy, the height difference between these values can be regarded as the crop height within the study area. To evaluate the accuracy of the optimal estimates, this study selected the 90th, 95th, and 99th percentiles as upper limits for crop height estimates. These percentiles represent the main canopy height, dominant canopy height, and extreme canopy height, respectively, and were used to assess the stability and sensitivity of crop height estimates under different percentile thresholds. Through comparative analysis, the optimal strategy for crop height prediction was determined (**Figure 5**). Comparative analysis was conducted to identify the optimal crop height prediction strategy.

### 2.8 Developing an enhanced evapotranspiration model based on UAV remote sensing and point cloud data

The FAO-56 dual crop coefficient method partitions crop evapotranspiration (ET_c_) into transpiration and soil evaporation components (Allen et al., 1998). The calculation formula is shown in Equation 5:

(5) $$ET_{c}=K_{c}*ET_{0}=\left({\text{K}}_{\text{s}}*{\text{K}}_{\text{cb}}+{\text{K}}_{\text{e}}\right){\text{ET}}_{0}$$

where K_cb_ is the basal crop coefficient, K_e_ is the soil evaporation coefficient, K_s_ is the water stress coefficient, and ET_0_ is reference evapotranspiration.

In the standard FAO-56 approach, the basal crop coefficient (K_cb_) is assigned fixed tabulated values for each growth stage (e.g., 0.15 for the initial stage, 1.10 for the mid-season stage, and 0.15 for the late-season stage for wheat). However, this static representation fails to capture the dynamic responses of crop transpiration to varying growth conditions, such as canopy development and plant water status. To address this limitation, the dual crop coefficient method was improved by replacing static parameters with UAV-derived dynamic crop variables. Specifically: (1) the dynamic K_cb_ was calculated using UAV-retrieved crop height and LAI; (2) the canopy cover fraction (f_c_), required for estimating the K_e_, was derived from LAI; and (3) K_s_ was computed based on the UAV-derived CWSI. Reference evapotranspiration (ET_0_) was estimated using the Penman-Monteith model: The calculation formula is shown in Equation 6:

(6) $$ET_{0}=\frac{0.408\Delta \left(R_{n}-G\right)+\frac{900u_{2}\left(e_{s}-e_{a}\right)}{T+273}}{\Delta +\text{γ}\left(1+0.34u_{2}\right)}$$

Where: ET_0_ is the reference crop evapotranspiration (mm); R_n_ is the net radiation flux (MJ m^-2^ day^-1^); G is the soil heat flux (MJ m^−2^ day^−1^); Δ is the slope of the saturation vapor pressure–temperature curve (kPa °C^−1^); γ is the psychrometric constant (kPa °C^−1^); T is the daily mean air temperature (°C); u_2_ is the wind speed at 2 m height (m s^−1^); e_s_ is the saturation vapor pressure (kPa); and e_a_ is the actual vapor pressure (kPa).

#### 2.8.1 Dynamic basal crop coefficient

Following the FAO-56 adjustment procedure, the tabulated K_cb_ values were first adjusted for local climatic conditions (Allen et al., 1998): The calculation formula is shown in Equation 7:

(7) $$K_{cb,adjust}=K_{cb,tab}+{\left(\frac{h}{3}\right)}^{0.3}\left[0.04*\left(u_{2}-2\right)-0.004\left(RH_{min}-45\right)\right]$$

The actual K_cb_ was then scaled by canopy density using LAI: The calculation formula is shown in Equation 8:

(8) $$K_{cb,a}=K_{c,min}+\left(K_{cb,adjust}-K_{c,min}\right)\left[1-\exp\left(-kLAI\right)\right]$$

The final K_cb_ accounting for water stress was calculated as shown in Equation 9:

(9) $$K_{cb}=K_{s}*K_{cb,a}$$

Among these, where K_cb_, adjust represents the basal crop coefficient corrected by meteorological factors during different growth stages; K_cb,tab_ represents the basal crop coefficient under standard conditions; u_2_ represents the daily average wind speed in m/s at 2 m above the crop canopy during different growth stages; RH_min_ represents the daily average minimum relative humidity in % during different growth stages; h represents the average plant height in m during different growth stages; when the basal crop coefficient is less than 0.45 under standard conditions, and meteorological correction is not required; K_cb, a_ indicates the basal crop coefficient corrected by LAI; k is the canopy attenuation coefficient, K = 0.7; K_c,min_ represents the minimum value for bare soil crop coefficient, the value being 0.15–0.20; ET_0_ represents reference crop evapotranspiration (i.e., evapotranspiration under standard meteorological conditions); K_s_ represents the crop stress coefficient K_s_.

#### 2.8.2 Soil evaporation coefficient and water stress coefficient

Evaporation from farmland soil occurs in the inter-row spaces or beneath the crop canopy. K_e_ reflects the short-term increase in soil evaporation intensity following rainfall or irrigation, which affects ET_c_. When the soil surface is relatively moist, K_e_ reaches its upper limit, K_c, max_. As the soil surface gradually dries, evaporation gradually declines. At this point, K_e_ is calculated using the following Equation 10:

(10) $$K_{e}=K_{r}\left(K_{c,max}-K_{cb}\right)\leq f_{ew}K_{c,max}$$

In the formula: K_c, max_ denotes the upper limit of K_c_, i.e., the maximum value of K_c_ after precipitation or irrigation; K_r_ represents the soil evaporation attenuation coefficient; few indicates the ratio of evaporating soil area to total soil area, i.e., the proportion of effective evaporation in the soil (%).

The calculation Equation 11 for K_c, max_ is:

(11) $$K_{c,max}=\max\{\{K_{cb,tab}+\left[0.04*\left(u_{2}-2\right)-0.004\left(RH_{min}-45\right)\right]{\left(\frac{h_{max}}{3}\right)}^{0.3}\},(K_{cb}+0.05)\}$$

In the formula, h_max_ represents the average canopy height (m) during the crop growth stage.

Soil evaporation can be divided into two stages: Stage 1 (energy-limited stage), during which soil moisture is relatively abundant and the evaporation reduction coefficient (K_r_) equals 1; and Stage 2 (falling-rate stage), during which soil moisture gradually decreases. When the cumulative evaporation depth (D_e_) is less than or equal to the readily evaporable water (REW), the calculation of the evaporation reduction coefficient (K_r_) follows established methods reported in the literature.

The calculation formula for f_ew_ in Equation 12 is:

(12) $$f_{ew}=min\left(1-f_{c},f_{w}\right)$$

In the formula, 1-f_c_ represents the average ratio of exposed soil; f_w_ denotes the proportion of soil moistened by precipitation.

The calculation formula for f_c_ in Equation 13 is:

(13) $$f_{c}={\left[\frac{K_{cb}-K_{c.min}}{K_{c,max}-K_{c,min}}\right]}^{\left(1+0.5h\right)}$$

K_s_ reflects the impact of insufficient soil moisture content in the root zone on crop transpiration. Jackson (1982) proposed the calculation formula for the CWSI, which indicates an inverse relationship between CWSI and the water demand of the crop under study. The crop coefficient (K_s_) can also be derived from CWSI using the following Equation 14:

(14) $$K_{s-CWSI}=1-CWSI$$

#### 2.8.3 Evaporation pan measurements for ET_0_ validation

To obtain measured reference crop evapotranspiration (ET_0_), a standard ADM7-type micro-evaporation pan with a diameter of 20 cm was used in this study. The pan was installed at the Xinxiang meteorological station in strict accordance with the Specifications for Surface Water Evaporation Observation (SL 630-2013). It was mounted on a wooden platform approximately 15 cm above the ground to ensure adequate ventilation, with the pan rim maintained in a horizontal position. Daily evaporation was measured at 08:00 local time using a hook gauge. The pan coefficient (K_pan_) was then calculated using an empirical formula recommended in FAO-56 and subsequently used to derive ET_0_._0_ (Gundekar et al., 2008).

Based on the fetch distance of 20 m of grass surrounding the meteorological station, the daily mean wind speed, and the daily mean relative humidity, this study selected the formula proposed by Snyder to calculate Kpan (Snyder, 1992). This Snyder formula accounts for the effects of fetch distance, wind speed, and relative humidity on the differences in energy and aerodynamic conditions between the evaporation pan and the standard reference crop, making it suitable for the actual conditions of the local irrigated area.

The resulting measured ET_0_ data were used to validate the ET_0_ values estimated by the Penman-Monteith model. The validation work covered the jointing, heading, and grain filling stages of the crop. By comparing the daily ET_0_ values obtained from the two methods and using statistical indicators including the coefficient of determination (R^2^), root mean square error (RMSE), and normalized root mean square error (nRMSE), the accuracy of the model estimates was evaluated.

### 2.9 Machine learning algorithms

To mitigate redundancy and multicollinearity in predicting winter wheat PMC, Pearson correlation analysis was used to select features with high correlation to PMC. This step, implemented with Pandas (v1.4.2) and Matplotlib (v3.5.1) in Python 3.9.12, reduced information redundancy and provided a robust data foundation for subsequent machine learning modeling.

Machine learning regression algorithms effectively capture both linear and nonlinear relationships between remote sensing data and crop parameters, often outperforming other methods in multivariable predictions. In this study, four algorithms—PLSR, BPNN, SVR, and RFR—were used to develop estimation models for wheat PMC. First, the fundamental concept of PLSR involves projecting both the independent variable space and the dependent variable space to identify a set of new composite variables (referred to as partial least squares components). These components aim to explain as much variation as possible in both the independent and dependent variables while achieving maximum correlation between them. This approach achieves dimensionality reduction and information extraction from the data, thereby establishing a regression model between the independent and dependent variables (Wu et al., 2025). It is a statistical method for solving multivariate linear regression problems, performing particularly well when independent variables exhibit multicollinearity and when sample sizes are relatively small. BPNN is a multi-layer feedforward network trained by minimizing the error between predicted and observed outputs through gradient descent. In this study, a three-layer BPNN architecture (input layer, one hidden layer, and output layer) was implemented using the Python Scikit-Learn library. The number of neurons in the hidden layer was optimized using Optuna. The activation function for the hidden layer was ReLU, and the output layer used a linear activation function for regression (Zhao et al., 2023). Secondly, SVR is based on the Support Vector Machine algorithm, incorporating a sensitivity loss function to transform the classification algorithm into a regression algorithm. Extensive research indicates that SVR performs well in crop index inversion and monitoring studies, making it suitable for small-sample learning. Model parameters are configured as follows: the kernel function uses the commonly employed radial basis function, the penalty factor C is set to 1.0, and the loss function Gamma is set to 1/n_Features (Zhang et al., 2020). Conversely, RFR is an ensemble machine learning algorithm based on multiple decision trees using bagging technology. This algorithm exhibits strong robustness against noise and overfitting; it effectively handles high-dimensional and collinear data while offering advantages such as fast training speed and no requirement for preprocessing input data (Kheir et al., 2025).

Hyperparameter tuning is critical for optimizing the performance of machine learning algorithms. Complex models with numerous parameters are prone to overfitting, whereas simpler models often struggle to capture complex nonlinear relationships and are therefore susceptible to underfitting. Optuna is an open-source hyperparameter optimization framework that is compatible with various machine learning libraries and enables efficient exploration of the hyperparameter search space (Akiba et al., 2019). In this study, we leverage Optuna’s Sequential Model-Based Optimization (SMBO) to efficiently search for optimal hyperparameters. Optuna continuously explores the parameter space by minimizing the value of the cost function to identify the best hyperparameter combination. Optuna’s advantages include efficiency, ease of use, scalability, and customizability. Building upon this foundation, we employed five-fold cross-validation and Optuna to find the optimal model hyperparameter combinations for each dataset used as the training set. The five-fold cross-validation approach involves dividing the training set into five subsets. Four subsets are cyclically used as training data, where Optuna experiments with different model hyperparameter combinations, while the remaining subset serves as validation data to evaluate the performance of each combination.

This study collected 180 samples at each growth stage. A PMC prediction model was constructed using canopy temperature data acquired by drones, highly correlated VIs, and improved ET values, all compared against ground-truth measurements. To evaluate the impact of different feature combinations on model performance, the experiment designed five input combinations incorporating VIs, ET, and TIs. Four distinct machine learning algorithms-PLSR, BPNN, SVR, and RFR-were assessed to construct PMC prediction models.

### 2.10 Evaluation metrics

The model performance was evaluated using three metrics: R^2^, RMSE, and nRMSE. The R^2^ value ranges from 0 to 1, with higher values indicating better model fitting and predictive accuracy. The calculation formula is shown in Equation 15: In contrast, lower values of RMSE and nRMSE indicate improved predictive performance of the model. The formulas for the two are Equation 16 and Equation 17, respectively:

(15) $$R^{2}=1-\frac{\sum_{i=1}^{n}{\left(x_{i}-y_{i}\right)}^{2}}{\sum_{i=1}^{n}{\left(x_{i}-\bar{y}\right)}^{2}}$$

(16) $$RMSE=\sqrt{\frac{\sum_{i=1}^{n}{\left(x_{i}-y_{i}\right)}^{2}}{n}}$$

(17) $$nRMSE=\frac{RMSE}{\bar{y}}\times 100\%$$

In the formula, *x_i_* represents the measured value; *y_i_* represents the estimated value; *i*, *j* are sample identifiers; *n* is the number of samples; $\bar{y}$ is the average value of the measured values.

**Figure 6:**
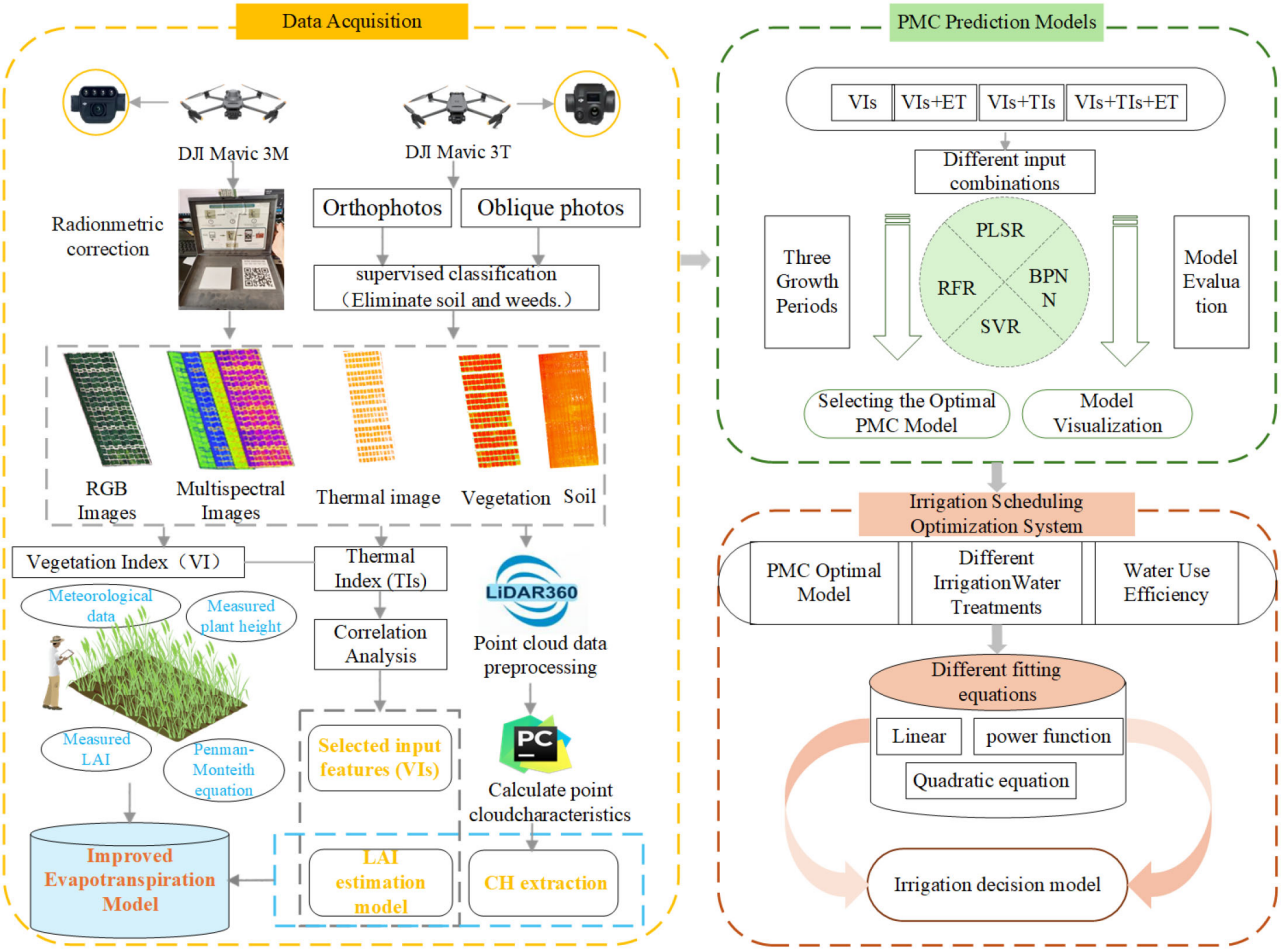
Technical workflow diagram of this experiment.

The overall workflow of this study, encompassing UAV data acquisition, ground truth collection, model development, and irrigation scheduling optimization, is illustrated in **Figure 6**.

## 3 Results

### 3.1 Statistical analysis of winter wheat plant moisture content under different water management treatments

**Figure 7:**
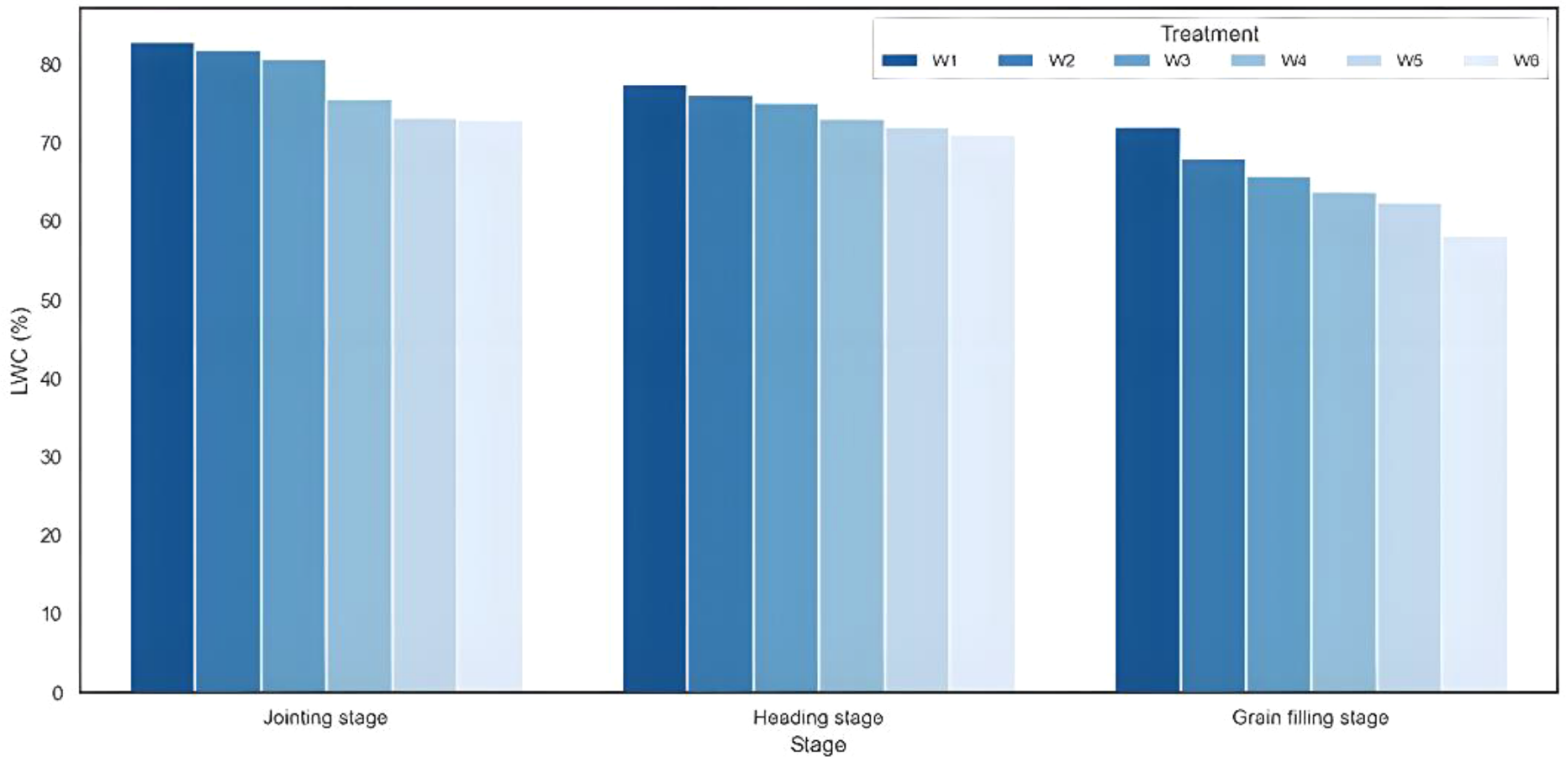
Distribution characteristics of PMC in wheat at different growth stages.

**Figure 7** illustrates the effects of different water treatments on PMC variations in winter wheat. As shown in **Figure 7**, PMC exhibited a declining trend throughout the growth period. Significant differences were observed among irrigation treatments at the same growth stage. Within the same growth stage, PMC increased progressively with higher irrigation volumes. The PMC values under different water treatments generally followed the order: W1 > W2 > W3 > W4 > W5 > W6. Additionally, from the jointing to grain filling stage in 2023, PMC decreased by 10.80% (W1 treatment), 13.94% (W2 treatment), 14.77% (W3 treatment), 11.82% (W4 treatment), 10.81% (W5 treatment), and 14.58% (W6 treatment), respectively. These results indicate that winter wheat PMC is closely correlated with water treatment levels. Therefore, estimating PMC can serve as a means to monitor winter wheat water status, which plays a crucial role in field irrigation management.

### 3.2 Estimation of field-scale evapotranspiration in winter wheat

During the jointing stage, LAI increased significantly. Subsequently, at the heading stage, LAI nearly reached its maximum value and gradually decreased during the later growth period. The overall trend followed by LAI aligns with the actual growth pattern of winter wheat.

**Figure 8:**
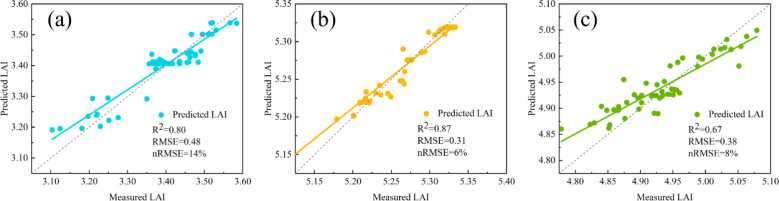
Regression scatter plots of LAI predicted values versus measured values based on the random forest model. **(A)** Jointing stage; **(B)** heading stage; **(C)** grain filling stage.

Using six vegetation indices—RDVI, OSAVI, VIopt, MNLI, SAVI, and NDVI—derived from multispectral images, a random forest model was developed to invert wheat leaf area index (LAI). The validation statistics of this model are presented in **Figure 8**. Results indicate that the LAI inversion model achieved R^2^ values ranging from 0.67 to 0.87 across three growth stages, with RMSE values between 0.31 and 0.48 and nRMSE values between 6% and 14%. The LAI inversion model achieved the highest accuracy during the heading stage, with R^2^ = 0.87, RMSE = 0.31, and nRMSE = 6%. Compared to the jointing and heading stages, model accuracy slightly decreased during the grain filling stage. LAI inversion results from all three growth stages were used to estimate ET, which was subsequently employed as a modeling variable for the winter wheat PMC prediction model.

**Figure 9:**
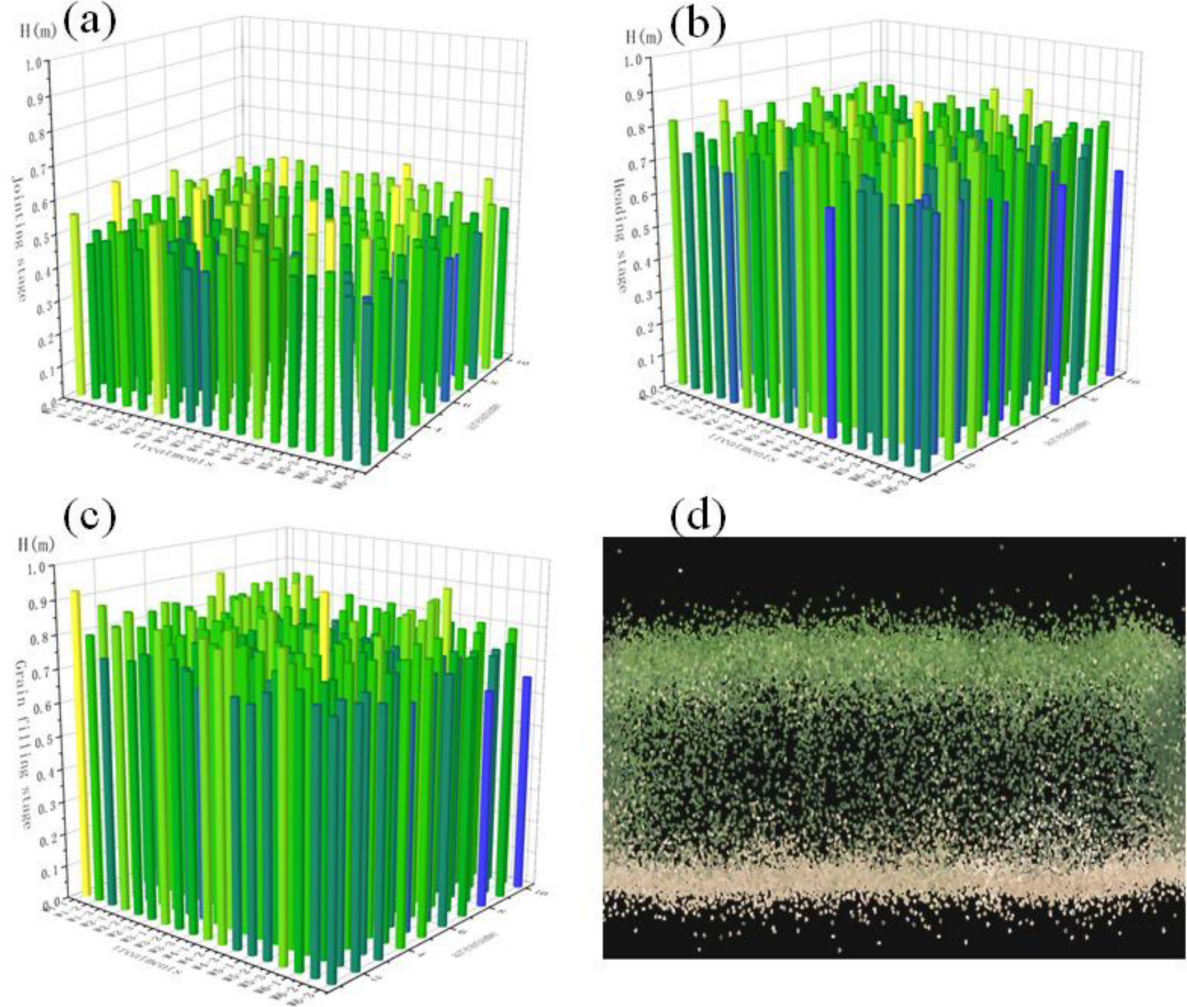
Crop height variables. **(A–C)** Crop height distribution **(D)** point cloud distribution within individual plots.

**Figure 9** shows the point cloud height distribution of a single wheat plot and the plant height distribution characteristics across three growth stages. As the growth stages progressed, crop height increased significantly. Then, during the grain filling stage, height reached its maximum value, and changes gradually diminished in the later growth stages.

**Table 3** presents the accuracy results of crop height estimation using the AIH method during the jointing, heading, and grain filling stages of winter wheat. The results indicate that the AIH method demonstrates good predictive accuracy across different growth stages. The R^2^, RMSE, and nRMSE values for different growth stages ranged from 0.63 to 0.77, 2.03 to 3.25 cm, and 3.26% to 5.05%, respectively. Among the three AIH methods for crop height estimation, the 95% AIH yielded the smallest error with R^2^ values ranging from 0.74 to 0.77. The highest R^2^ value of 0.77 was observed during the heading stage, followed by the jointing and grain filling stages. Concurrently, the heading stage exhibited the smallest RMSE and nRMSE values at 2.03 cm and 3.26%, respectively.

**Table 3:** Prediction accuracy of crop height estimates using different AIH methods.

Growth stages	AIH_90th			AIH_95th			AIH_99th		
	R^2^	RMSE(cm)	nRMSE(%)	R^2^	RMSE(cm)	nRMSE(%)	R^2^	RMSE(cm)	nRMSE(%)
Jointing stage	0.63	2.54	5.05	0.76	2.03	4.03	0.74	2.16	4.03
Heading stage	0.66	3.03	4.01	0.77	2.47	3.26	0.73	2.69	3.56
Grain filling stage	0.64	3.25	4.14	0.74	2.76	3.51	0.72	2.84	3.62

**Figure 10:**
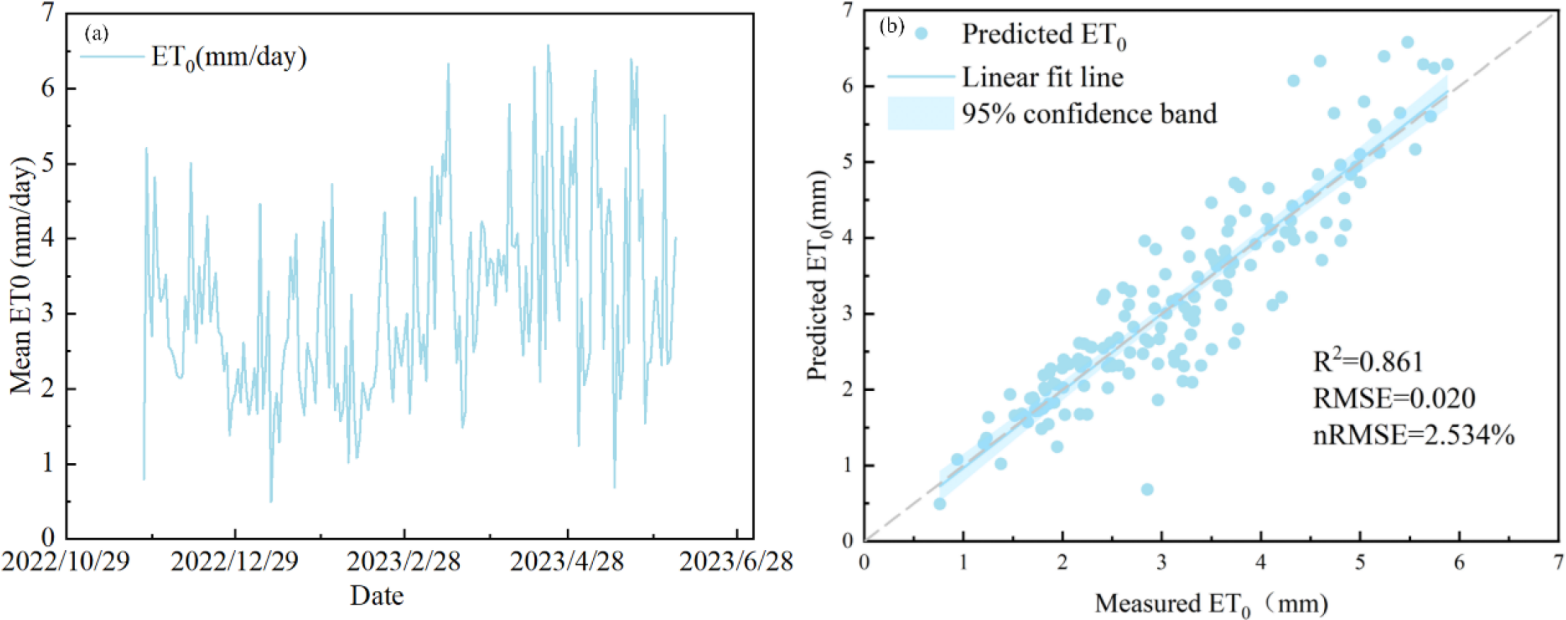
The Penman-Monteith model calculations and the measured crop potential ET_0_, evapotranspiration from evaporation pans for different growth stages of winter wheat. The dashed lines indicate the expected 1:1 relationship.

**Figure 10** shows the comparison results between the Penman-Monteith model calculations and the measured crop ET_0_ from evaporation pans for different growth stages of winter wheat. Comparative analysis indicates that ET_0_ calculated using the Penman-Monteith model shows good agreement with measured evaporation from a 20 cm evaporation pan (R^2^ = 0.861). The low root mean square error (RMSE = 0.020 mm d^−1^) and normalized root mean square error (NRMSE = 2.534%) further confirm the reliability of the model estimates, indicating its ability to accurately characterize actual ET conditions under the study conditions.

Based on the above-mentioned leaf area index and plant height inversion results, combined with the P-M equation and dual crop coefficients, a dynamic estimation model for field-scale ET in winter wheat fields was established. Based on published literature (Zhao et al., 2013), this study determined the static base crop coefficient (K_cb_) for each growth stage of winter wheat: 0.25 for the initial stage and soil freezing period, 1.15 for the mid-stage (jointing to heading), and 0.30 for the harvest stage. Using these coefficients to construct a static model for ET estimation, the results showed high consistency with dynamic model simulations in overall trends. The average coefficient of determination (R^2^) for the comparison across the jointing, heading, and grain filling stages was 0.724, indicating that both methods captured the overall ET trend consistently.

Specific accuracy metrics for each growth stage are presented in **Table 4**, Accuracy of ET Estimation at Different Growth Stages Between Models Based on Static Kcb Coefficients and Dynamic Models. The model performed best during the heading stage (R^2^ = 0.776, nRMSE = 6.47%), while the relative error was highest during the jointing stage (nRMSE = 15.95%).

**Table 4:** Accuracy of ET estimation at different growth stages between models based on static K_cb_ coefficients and dynamic models.

Growth stages	R^2^	RMSE(mm/day)	nRMSE(%)
Jointing stage	0.751	0.409	15.95
Heading stage	0.776	0.240	6.47
Grain filling stage	0.646	0.235	6.18

**Figure 11:**
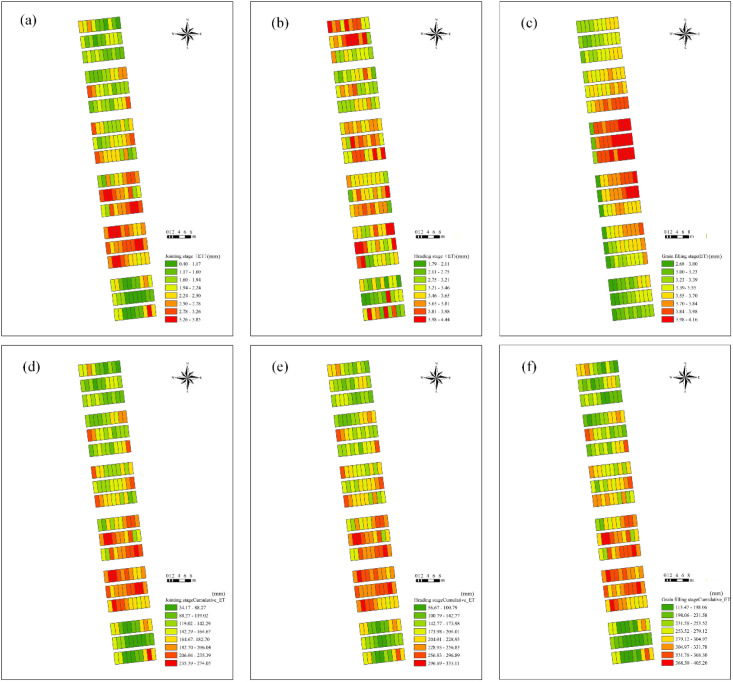
Spatial distribution maps of actual crop ET and cumulative ET. **(A–C)** show actual ET; **(D–F)** show cumulative ET.

During the early growth stage, ET_c_ was relatively low. Subsequently, ET_c_ increased with some relative fluctuations within a certain range, gradually decreasing toward the later stages. Estimated values of ET_c_ and cumulative ET_c_ during the three growth stages of winter wheat, along with the spatial distribution of cumulative ET_c_, are shown in **Figure 11**. The ET for winter wheat ranged from 0.401 to 3.851 mm during the jointing stage, 1.792 to 4.444 mm during the heading stage, and 2.683 to 4.162 mm during the grain filling stage. The cumulative ET over the three growth stages ranged from 31.178 to 274.049 mm, 56.678 to 333.107 mm, and 113.474 to 405.195 mm, respectively. These results are consistent with previously reported observations from similar climatic regions and agronomic conditions both domestically and internationally, thereby confirming the reliability of the proposed estimation method (Tang et al., 2018).

### 3.3 VIs, TIs, and ETs and their correlation analysis with PMC

**Figure 12:**
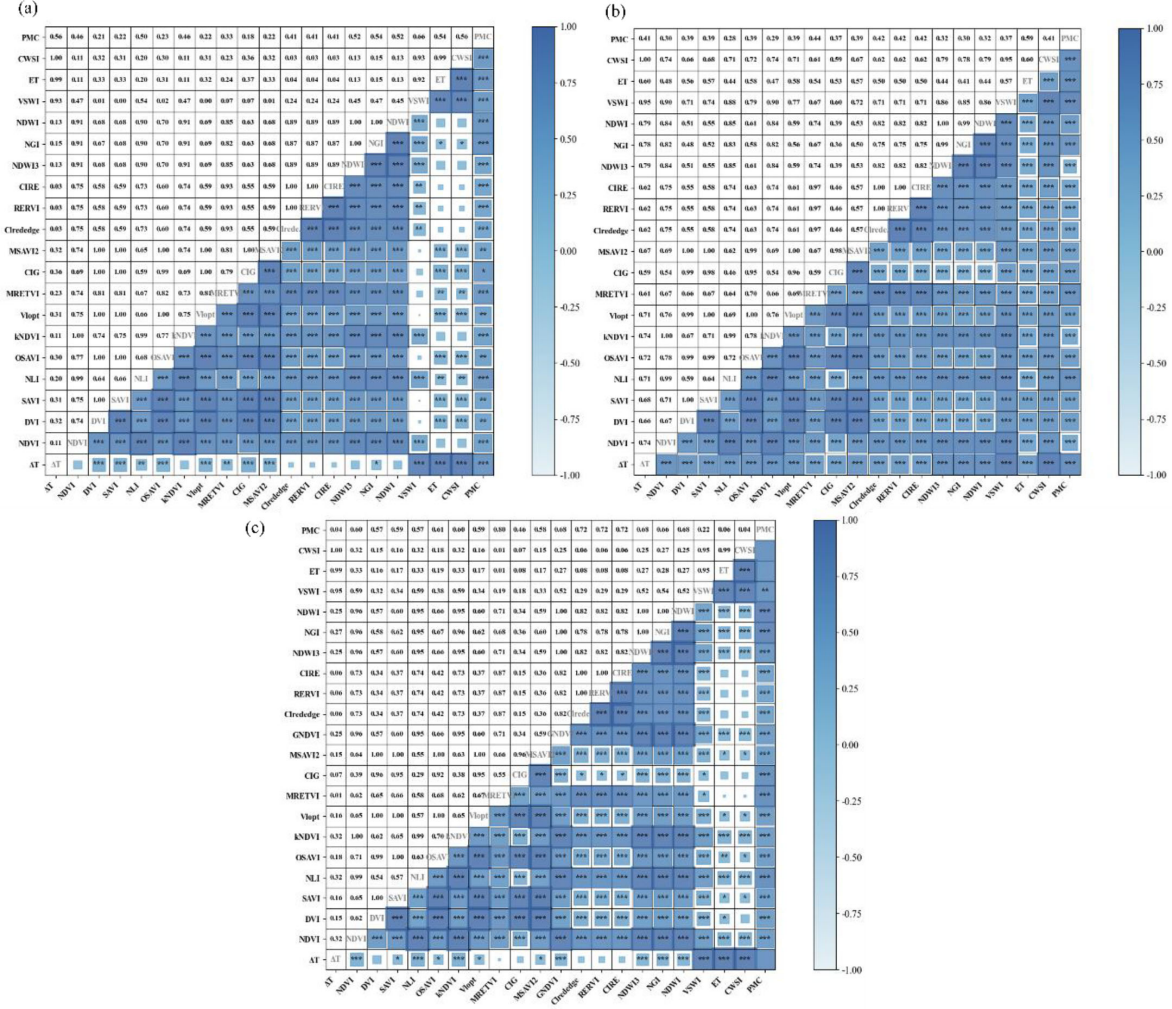
Absolute values of pearson correlation coefficients (|r|) between VIs, TIs, ET, and PMC at different growth stages of wheat. **(A)** Jointing stage; **(B)** heading stage; **(C)** grain filling stage.

**Figure 12** displays the Pearson correlation coefficient matrix between VIs, TIs, ET, and PMC at different growth stages of winter wheat. To facilitate clearer comparison of correlations, the absolute values of correlation coefficients (|r|) were calculated. During the jointing stage, Pearson correlation coefficients related to PMC ranged from 0.513 to 0.738. At the heading stage and grain filling stage, the ranges were 0.314–0.866 and 0.485–0.881, respectively. Based on Pearson correlation coefficients, six VIs with the best overall performance across the three growth stages were selected: NGI, CIG, VSWI, NDWI, MRETV, and NLI. Subsequently, by combining these with TIs (ΔT, CWSI) and ET, four machine learning algorithms—RFR, BPNN, SVR, and PLSR—were employed to develop winter wheat PMC inversion models for different growth stages.

### 3.4 Prediction model for winter wheat PMC and WUE

Four distinct machine learning algorithms were employed to estimate winter wheat PMC (**Table 5**). Results indicate varying estimation accuracies across different growth stages of winter wheat. The RFR algorithm yielded an average R^2^ of 0.778 and an average RMSE of 0.042; the BPNN algorithm produced an average R^2^ of 0.534 and an average RMSE of 0.059; The PLSR algorithm yielded an average R^2^ of 0.613 and an average RMSE of 0.054. The SVR algorithm achieved an average R^2^ of 0.599 and an average RMSE of 0.056. The estimation accuracy of all four models for winter wheat PMC progressively improved with crop growth, reaching the highest model accuracy during the grain filling stage.

**Table 5:** Accuracy of wheat PMC estimation using different feature combinations and machine learning algorithms throughout the growth cycle.

Machine learning	Growth stages	Jointing stage			Heading stage			Grain filling stage		
	Features	R^2^	RMSE	nRMSE	R^2^	RMSE	nRMSE	R^2^	RMSE	nRMSE
				(%)			(%)			(%)
PLSR	VIs	0.608	0.097	11.532	0.597	0.033	4.304	0.619	0.034	5.285
	ET	0.534	0.103	12.296	0.546	0.036	4.631	0.587	0.034	4.425
	VIs+ET	0.609	0.097	11.513	0.662	0.030	3.986	0.620	0.034	5.284
	VIs+TIs	0.610	0.096	11.495	0.621	0.032	4.138	0.636	0.033	5.168
	Vis+TIs+ET	0.615	0.096	11.432	0.664	0.030	3.886	0.673	0.031	4.960
BPNN	VIs	0.482	0.109	12.956	0.496	0.044	5.725	0.519	0.038	5.947
	ET	0.455	0.112	13.364	0.397	0.042	5.353	0.498	0.040	6.227
	VIs+ET	0.552	0.099	11.759	0.543	0.036	4.658	0.561	0.036	5.666
	VIs+TIs	0.540	0.103	12.242	0.518	0.037	4.780	0.619	0.034	5.310
	Vis+TIs+ET	0.607	0.094	11.238	0.594	0.034	4.399	0.630	0.033	5.221
SVR	VIs	0.518	0.105	12.483	0.597	0.033	4.304	0.653	0.032	5.047
	ET	0.449	0.112	13.365	0.546	0.036	4.631	0.587	0.034	4.425
	VIs+ET	0.524	0.104	12.385	0.664	0.030	3.916	0.692	0.030	4.754
	VIs+TIs	0.524	0.104	12.415	0.621	0.032	4.138	0.692	0.030	4.755
	Vis+TIs+ET	0.530	0.104	12.423	0.672	0.030	3.886	0.721	0.029	4.523
RFR	VIs	0.659	0.089	10.574	0.768	0.026	4.139	0.803	0.023	3.018
	ET	0.522	0.105	12.477	0.645	0.034	5.258	0.679	0.030	3.834
	VIs+ET	0.800	0.068	8.102	0.852	0.021	3.296	0.859	0.020	2.563
	VIs+TIs	0.780	0.072	8.574	0.835	0.022	3.478	0.856	0.020	2.589
	Vis+TIs+ET	0.844	0.059	7.089	0.861	0.020	2.534	0.900	0.017	2.688

VIs, ET, and TIs refer to the multispectral vegetation index, ET, and temperature index, respectively.

**Figure 13:**
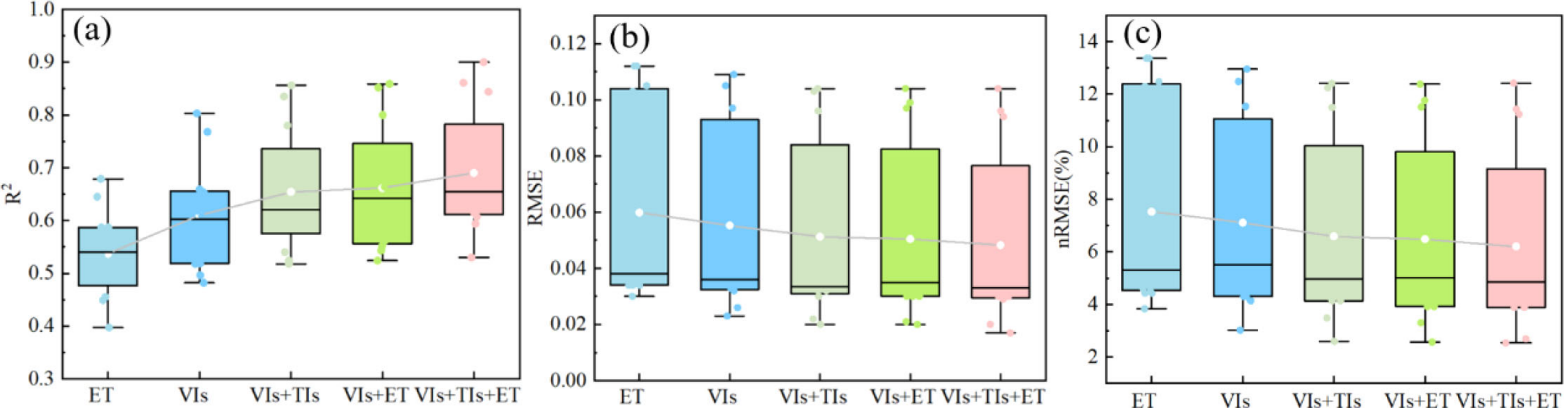
Accuracy results of different model combinations across three growth stages. **(A)** Jointing stage; **(B)** heading stage; **(C)** grain filling stage.

In the models constructed using multivariate combinations (**Figure 13**), the estimation accuracy of the VIs+ET or VIs+CWSI combinations showed some improvement compared to models based solely on VIs. Compared to the estimation accuracy of VIs+ET or VIs+CWSI, the model constructed using the VIs+ET+CWSI combination demonstrated the best performance in predicting PMC, reaching its peak during the grain filling stage with an R^2^ of 0.900 and an RMSE of 0.017, while the nRMSE was 2.688%.

**Figure 14:**
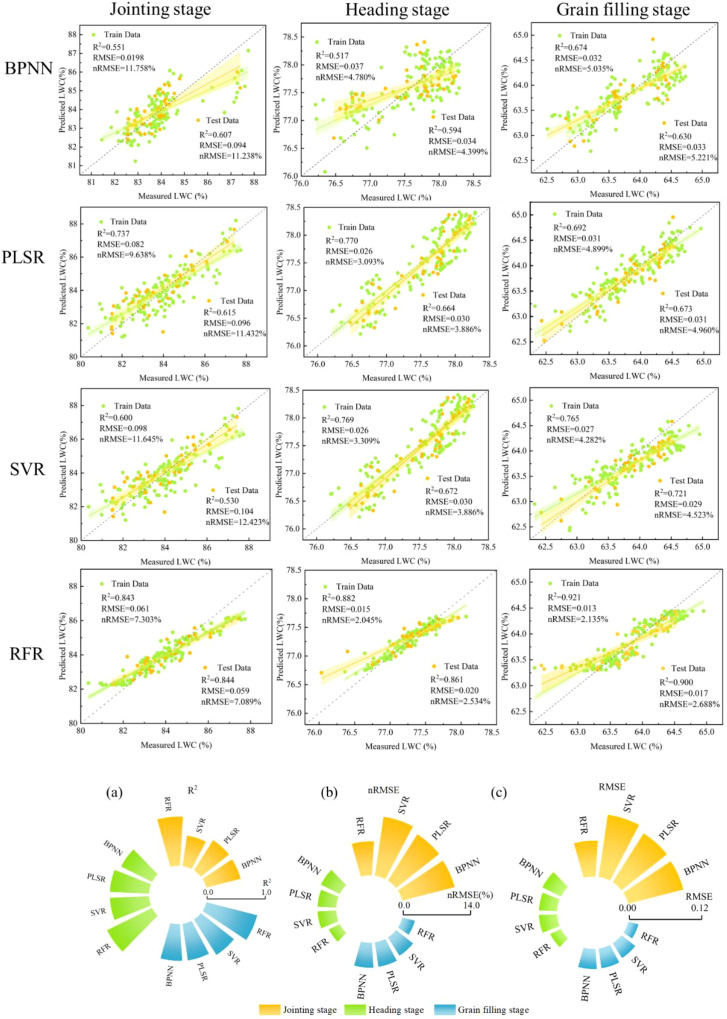
Estimation accuracy of PMC based on the combination of VIs+ET+CWSI across four machine learning algorithms during three growing seasons. **(A)** Jointing stage; **(B)** heading stage; **(C)** grain filling stage.

Compared to BPNN, PLSR, and SVR models, the model constructed using the RFR algorithm demonstrated overall superior performance (**Figure 14**). It consistently exhibited high accuracy across all three growth stages, with R^2^ values exceeding 0.522 throughout the entire growth period, RMSE values below 0.105, and nRMSE values under 12.477%. Particularly during the grain filling stage, the model achieved its highest precision.

To further determine the optimal WUE, combined with the estimated PMC at different growth stages from the winter wheat PMC estimation model, three different fitting models were employed to derive the corresponding PMC thresholds for optimal WUE at each growth stage. Water stress induced by different water treatments significantly affected the WUE of winter wheat. The fitting results for PMC and WUE were relatively good during the jointing and heading stages, but lower during the grain filling stage (**Table 6**).

**Table 6:** Three fitting methods for PMC and WUE under different water treatments.

Growing stage	Treatment	Linear	Linear_R^2^	Quadratic	Quadratic_R^2^	Power	Power_R^2^
Jointing stage	W1	y = -0.1706x + 15.2916	0.632	y = 0.0041x^2^ -0.8562x + 44.2271	0.634	y=0.0733x^-14.6054	0.609
	W2	y = -0.0568x + 5.5930	0.640	y = -0.0164x^2^ + 2.6786x -108.2794	0.700	y=0.0079x^-12.4332	0.624
	W3	y = -0.1970x + 17.3796	0.669	y = -0.0713x^2^ + 11.6638x -475.5100	0.742	y=0.0559x^-15.4738	0.624
	W4	y = -0.1275x + 11.6428	0.406	y = -0.0806x^2^ + 13.1537x -535.6116	0.765	y=0.2399x^-7.9423	0.342
	W5	y = -0.1554x + 13.8922	0.597	y = -0.0584x^2^ + 9.5623x -390.0395	0.662	y=0.0919x^-12.6882	0.566
	W6	y = -0.0374x + 3.7981	0.340	y = 0.0319x^2^ -5.5385x + 240.9425	0.540	y=0.2305x^-6.0338	0.359
Heading stage	W1	y = -0.2103x + 17.8917	0.493	y = -0.1852x^2^ + 28.4444x -1090.3236	0.603	y=0.1348x^-9.6781	0.475
	W2	y = -0.1627x + 14.0713	0.418	y = -0.2331x^2^ + 35.9703x -1386.3881	0.609	y=0.3206x^-5.3870	0.402
	W3	y = -0.2162x + 18.2072	0.797	y = -0.1699x^2^ + 26.0834x -999.6933	0.850	y=0.0821x^-11.2546	0.786
	W4	y = -0.1496x + 13.0820	0.485	y = -0.2336x^2^ + 35.9450x -1381.0022	0.823	y=0.2352x^-7.2336	0.461
	W5	y = -0.1765x + 15.0917	0.606	y = 0.1505x^2^ -23.4696x + 916.0747	0.696	y=0.0466x^-8.1973	0.620
	W6	y = -0.0483x + 5.2073	0.205	y = -0.1261x^2^ + 19.4968x -752.3179	0.539	y=0.0309x^-9.0556	0.199
Grain filling stage	W1	y = 0.0400x –0.4368	0.320	y = -0.0837x^2^ + 10.7184x -340.8935	0.687	y=3.6273x^1.1993	0.320
	W2	y = -0.4127x + 28.4541	0.564	y = 0.1418x^2^ -18.4759x + 603.7337	0.585	y=0.3206x^-5.3870	0.577
	W3	y = 0.0097x +1.3272	0.009	y = 0.1620x^2^ -20.6590x + 660.5014	0.596	y=2.2459x^0.3155	0.009
	W4	y = -0.1053x + 8.5448	0.728	y = -0.0194x^2^ + 2.3534x -69.4975	0.740	y=0.3652x^-3.5787	0.722
	W5	y = -0.2261x + 16.2835	0.432	y = 0.3061x^2^ -39.3554x + 1266.5722	0.608	y=0.1181x^-9.7237	0.451
	W6	y = -0.2521x + 17.9005	0.356	y = 0.3667x^2^ -47.1005x + 1514.0905	0.528	y=0.7730x^-2.5016	0.375

y represents the threshold for winter wheat water use efficiency, while x denotes the corresponding plant moisture content for each model.

**Figure 15:**
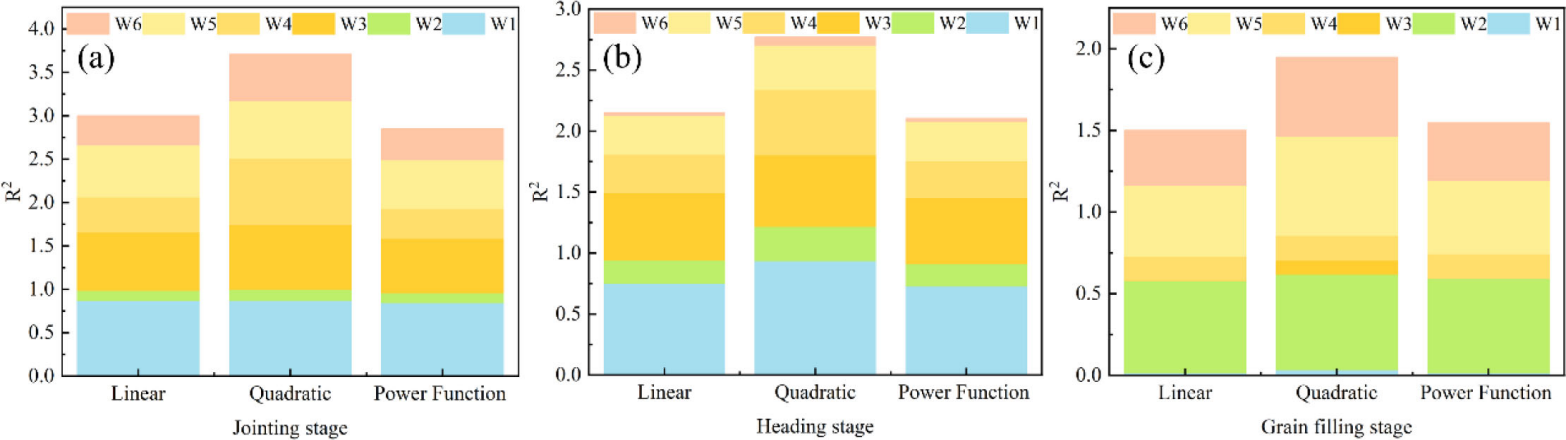
Fitting results for PMC and WUE under three conditions. **(A)** Jointing stage; **(B)** heading stage; **(C)** grain filling stage.

As shown in **Figure 15**, the WUE models for winter wheat based on quadratic regression methods at different growth stages exhibit single parameters, high accuracy, and strong practicality. Under the quadratic fitting equations, optimal WUE values were obtained for various growth stages.

During the jointing stage of irrigation treatments, the quadratic model W3 treatment achieved the best WUE, with a PMC threshold of 81.8%. During the heading and grain filling stages, the W1 treatment yielded the highest WUE, with PMC thresholds of 76.8% and 64.0%, respectively. Irrigation volumes can be adjusted based on the PMC thresholds for different growth stages to ensure optimal WUE throughout the jointing, heading, and grain filling periods of winter wheat.

### 3.5 Spatio-temporal distribution of wheat PMC

**Figure 16:**
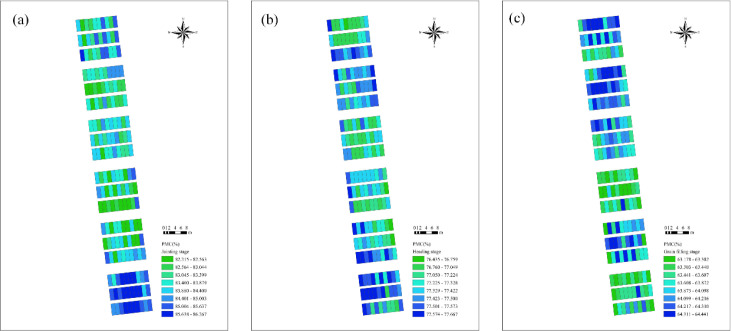
Spatial distribution map of PMC. **(A)** Jointing stage; **(B)** heading stage; **(C)** grain filling stage.

**Figure 16** presents the winter wheat PMC values predicted by the RFR model using the VIs+ET+TIs combination, illustrating the spatial distribution of PMC across different growth stages under various water treatments. Within the same growth stage, PMC values varied among plots, which were closely related to the water treatment applied. During the jointing stage, winter wheat PMC remained at a relatively high level. At this vegetative growth phase, the crop undergoes rapid growth and requires substantial water to support cell division and expansion, as well as the formation of new tissues and organs. A considerable proportion of water exists as free water, leading to strong transpiration activity and consequently high PMC values. As the growth cycle progresses, differences in PMC among plots under different water treatments gradually emerge and become particularly pronounced during the grain filling stage. This pattern indicates that water stress and increased crop water consumption jointly influence PMC dynamics. Overall, winter wheat PMC at different growth stages exhibits distinct variations depending on the water supply level.

### 3.6 Importance of model features

**Figure 17:**
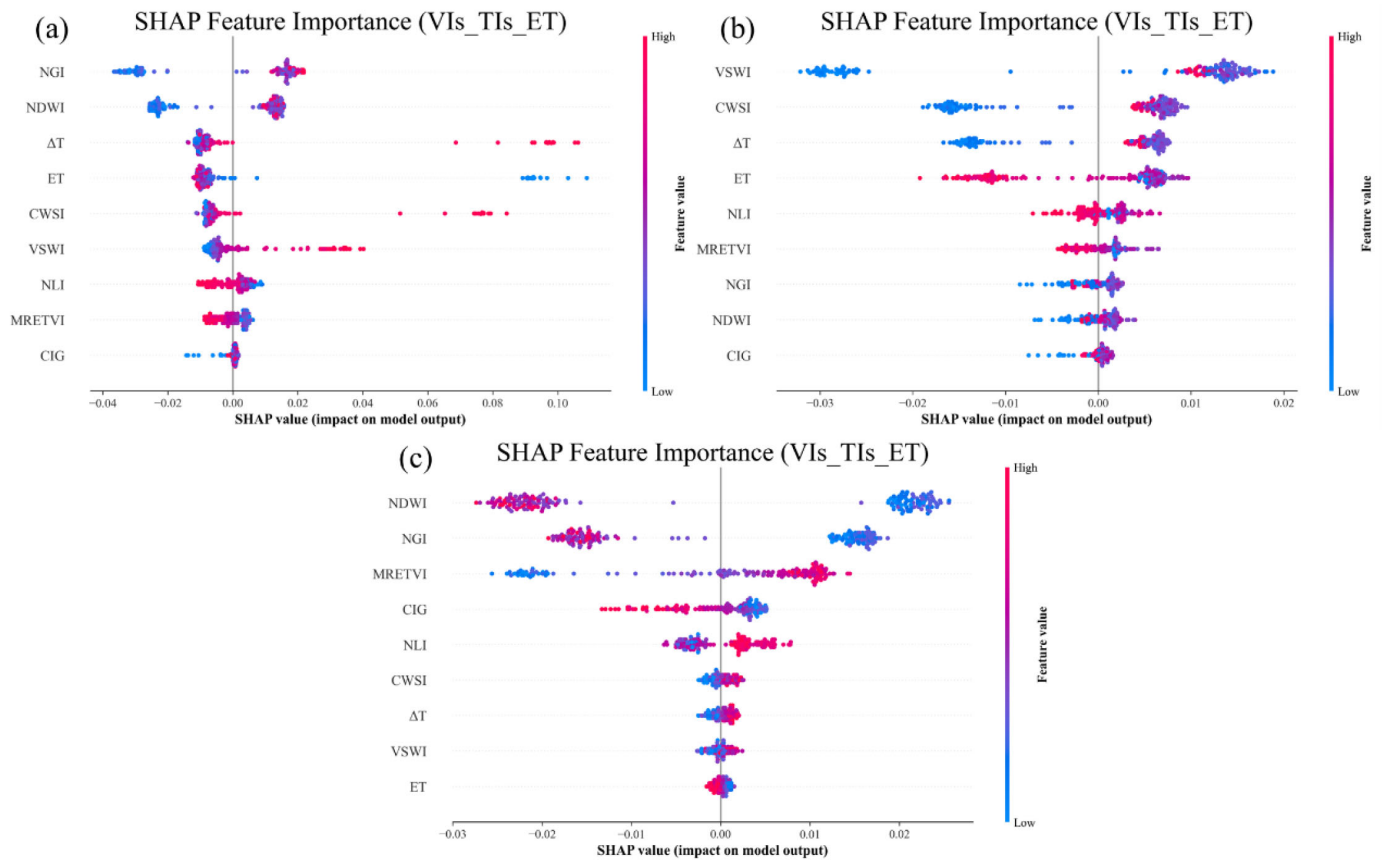
SHAP plots for the random forest model using the feature combination of VIs+ET+TIs. **(A)** Jointing stage; **(B)** heading stage; **(C)** grain filling stage.

To further elucidate the behavioral characteristics of each model and evaluate the relative contributions of selected features, the VIs+ET+TIs combination from the optimal (RFR) model was subjected to SHapley Additive exPlanations (SHAP) visualization analysis (**Figure 17**), thereby assessing the importance of each feature in PMC prediction. The results reveal that VIs, canopy temperature, and ET are pivotal factors in PMC estimation during the jointing to heading stages. VIs played a substantial role in the predictive model by directly capturing the spectral characteristics of plants. ET and canopy temperature complemented the model by reflecting subtle variations in plant water stress and canopy structure. TIs (ΔT, CWSI) and ET made notable contributions to predicting PMC during the jointing and heading stages, which correspond to healthy, high-water-content plant conditions, thus providing essential supplementary information for tracking water fluctuations. The NGI and NDWI contributed most significantly to the RFR model during the jointing stage, while VSWI showed a notable positive correlation with the model during the heading stage, indicating that VSWI values increased substantially when PMC was high. During the grain filling stage, NGI and NDWI exhibited significant negative correlations with the model, wherein higher index values exerted a stronger inhibitory effect on PMC. This shift can be explained by underlying crop physiological mechanisms. As leaves progressively senesce, chlorophyll degradation leads to a decline in NGI, which consequently becomes negatively correlated with PMC. Concurrently, NDWI exhibits heightened sensitivity to water stress. Because plants preferentially allocate moisture to grain development over vegetative tissues, leaf water content decreases, driving PMC downward and causing the relationship between NDWI and PMC to shift from positive to negative. Collectively, these changes reflect the physiological transition from vegetative to reproductive growth. Furthermore, the contributions of TIs and ET were relatively minor during this stage, primarily because leaf transpiration diminishes with progressive senescence, thereby weakening the correlation between canopy thermal indices and PMC. Nonetheless, these indices provide valuable multi-source information for accurately estimating winter wheat PMC across various growth stages.

## 4 Discussion

### 4.1 Variations in prediction accuracy of moisture content in crop plants at different growth stages

Winter wheat is a water-sensitive crop. Water deficiency during the jointing stage, heading stage, and grain filling stage can slow growth and development, cause leaf wilting, and even reduce yields (Tari, 2016). Consistent with reported findings, Zhang et al. (2024) identified the jointing stage as the most sensitive growth phase to water stress, followed by the heading stage and grain filling stage. This is primarily because, as crops grow, the canopy gradually becomes denser, leading to a saturation effect in VIs calculated from spectral information. That is, the information on canopy uptake capacity gradually saturates and diminishes. Conversely, during the jointing stage, the correlation between ET and PMC is weaker, while the correlation between VIs and PMC is stronger (Wang et al., 2024). This occurs because in the early growth stages, canopy coverage is low and leaf area is small. Thus, the warming effect caused by reduced canopy transpiration due to water deficiency is not significant, and PMC values in irrigated treatments tend to be similar, diminishing the temperature index’s ability to reflect PMC. Conversely, during the heading stage, as LAI and plant height increase, the warming effect from reduced canopy transpiration due to water deficiency becomes more pronounced, leading to stronger correlations between ET and TIs with PMC. Conversely, during the grain filling stage, as wheat undergoes physiological aging and a rapid decline in leaf physiological activity, canopy structure may fail to capture these dynamics, directly reducing the temperature index’s sensitivity to PMC variations. However, incorporating ET’s physical characteristics in the late growth stage only marginally improves model accuracy. This is because crop water dynamics primarily concentrate in the grain during this phase, causing canopy structure to exhibit distinct conditions compared to other growth stages, thereby diminishing the influence of ET-induced structural features (Peng et al., 2021). Therefore, predictive model development should consider adopting approaches consistent with selecting growth-stage-based water stress indicators or segmented modeling; that is, assigning distinct water stress indicators to different crop growth stages. For instance, utilizing drones to achieve optimal solutions for the best estimation of crop water status represents a promising solution.

### 4.2 Performance enhancement of field-scale evapotranspiration prediction models

ET is the process by which liquid water vaporizes into the gaseous state at soil (or water) surfaces and within plant tissues, subsequently transferring into the atmosphere. It is intrinsically linked to crop physiological activities. As a factor appearing in both surface energy and water balances, ET serves as a vital link connecting ecological and hydrological processes, and is a key component in formulating farmland irrigation plans (Tang et al., 2019). The FAO dual crop coefficient method, widely used in developing precision agricultural irrigation plans, separates crop transpiration and soil evaporation. However, its accuracy remains significantly improvable. The baseline crop coefficient in the FAO-56 dual crop coefficient method is a static value, whereas crop ET is inherently a dynamic process. Variations in crop growth environments and vigor can cause temporal and spatial fluctuations in ET (Irmak et al., 2024). Therefore, the modified ET model developed in this study comprehensively considers the dynamic effects of factors such as LAI, plant height, and meteorological conditions on crop transpiration and soil evaporation.

As a critical growth parameter for crops, the extraction of crop height has been revolutionized by oblique photography technology compared to orthophotos, which are limited to capturing images solely from vertical angles. By planning flight paths and tilting cameras to specific angles, oblique photography comprehensively captures target imagery. Characterized by extensive coverage, high precision, and high resolution, it vividly presents crop texture, location, height, and other information, making it highly favored by surveying professionals (Lei et al., 2022). Previous studies have widely adopted the Canopy Height Model (CHM) for crop height extraction. However, comparative analyses of accuracy among different crop height extraction methods remain limited. Issues such as image matching, wheat sparsity, high-resolution crop DSM imagery, and bare soil effects may compromise the accuracy of average-based crop height extraction methods. This aligns with findings reported by Wang et al. (2023). With continuous improvements in point cloud accuracy, Crop Height estimation algorithms based on 3D point clouds are gaining increasing attention. The AIH method normalizes vegetation point clouds against absolute elevation. The resulting AIH values are then utilized as crop height for wheat, mitigating the impact of wheat density variations. Different AIH methods exhibit varying performance in crop height extraction. Li et al. (2024) extracted VIs and structural features from optical imagery, zenith and oblique photography, and LiDAR point cloud data. They employed disparity-optimized models such as RFR, Light Gradient Boosting Machine (LightGBM), Gradient Boosting Decision Tree (GBDT), and SVR to estimate corn aboveground biomass. Results indicated that AIH99 demonstrated higher estimation accuracy for Crop Height. This study further confirms that AIH methods exhibit greater accuracy compared to CHM methods. This contrasts with findings from Lu et al. (2021), potentially attributable to their focus on extracting crop height for summer maize.

The dual crop coefficient method incorporates dynamic plant height extracted from 3D point clouds to calculate the base crop coefficient. Simultaneously, it utilizes dynamic LAI derived from VIs to estimate canopy coverage within the soil evaporation coefficient, enabling accurate estimation and differentiation of winter wheat ET. LAI serves as a crucial parameter for evaluating crop canopy structure and function, reflecting crop growth status, and is widely applied in assessing photosynthesis, transpiration, and energy balance (Yang et al., 2025). Its magnitude significantly influences the distribution of net radiation between the soil surface and crop canopy, with net radiation being the primary source of energy for evaporation and transpiration. Since the FAO-56 dual crop coefficient method does not account for the effects of dynamic LAI and plant height on ET, this study incorporates both LAI and plant height in its calculations. A dual crop coefficient ET model was developed for estimating winter wheat ET in this region. Further research is needed to refine the dual crop coefficient model across different soil types and spatial scales.

### 4.3 Improving the performance of predictive models based on multimodal data and various machine learning approaches

Appropriate VIs are crucial for constructing PMC spectral monitoring models. However, the canopy structure and growth environment of winter wheat undergo continuous changes throughout its growth period. Particularly during the late growth stage under high vegetation cover, spectral saturation leads to variations in spectral reflectance data, making it challenging to accurately construct water prediction models for the entire growth period using a single vegetation index model (Xuan et al., 2024). A robust relationship between physiological-ecological indicators and spectral information is key to rapidly and non-destructively monitoring coupled multi-source data. Currently, multi-source data are widely applied in multispectral monitoring. However, most studies utilize a single type of multi-source data to build models independently or in combination, with few investigations discussing the impact of coupled multi-source data incorporating physical factors on the monitoring performance of winter wheat PMC models.

ET is the only component appearing in both the surface energy balance and water balance. It plays a crucial role in both the energy and water cycles and serves as a vital link connecting ecological and hydrological processes. ET and canopy temperature are closely related to PMC (Cui et al., 2024). CWSI also serves as a remote sensing indicator for assessing crop water status. Dynamic monitoring of CWSI variations enables real-time quantification of crop water stress levels, consistent with findings by Liu et al. (2025). Relying solely on canopy temperature histograms obtained from UAVs, CWSI significantly simplifies calculations, making it more suitable for large-scale farmlands with complex irrigation scenarios. Previous studies indicate (Aixia et al., 2022) that winter wheat PMC exhibits instability, with leaf area and plant height undergoing continuous growth during the jointing stage, particularly under varying water treatments. Ultimately, since ET and CWSI are primarily regulated by ecological factors, ET, ΔT, and CWSI serve as sensitive indicators at this stage. However, wheat plants enter the senescence phase during grain filling. Compared to the jointing stage, leaf photosynthetic capacity significantly declines during grain filling. Moreover, during senescence, photosynthetic performance becomes susceptible to varying irrigation regimes, thereby influencing ET. Conversely, as plant-wide PMC decreases, transpiration rates rapidly diminish. Low transpiration rates temporarily elevate canopy temperature, subsequently prompting rapid adjustments in canopy CWSI. When the PMC in wheat falls below the threshold required to sustain physiological activity, photosynthetic capacity is rapidly downregulated. Prolonged low PMC impairs chlorophyll biosynthesis and accelerates its degradation, resulting in leaf yellowing. Fluctuations in these sensitive physiological parameters lead to substantial variations in leaf area, which exhibits varying degrees of reduction. These findings are consistent with those reported by (Peng et al., 2023); Wang et al. (2023).

Compared to the accuracy achieved using vegetation indices alone, the incorporation of ET and TIs as features significantly enhances the model’s predictive accuracy, demonstrating marked superiority over conventional single-type vegetation index inversion models. Among the model components, ET and the CWSI played crucial roles, primarily due to their implementation of a dual-dimensional diagnosis of crop water status—namely, “instantaneous stress (ET) combined with cumulative deficit (CWSI).” Both ET and CWSI contain valuable information relevant to crop water prediction. The predictive model combining VIs, ET, and CWSI (VIs+ET+CWSI) achieved the best performance, further confirming the importance and potential of integrating multimodal data for diagnosing crop leaf water status. Different machine learning algorithms also exhibit varying degrees of performance in predicting winter wheat PMC. This study evaluated the performance of four machine learning regression algorithms—RFR, BPNN, PLSR, and SVR—in estimating PMC, with RFR demonstrating superior performance compared to all other models. Compared to BPNN and PLSR, RFR effectively mitigates overfitting risks and enhances model generalization by constructing multiple decision trees and averaging their predictions, thereby achieving higher accuracy. This discrepancy stems from the complex nonlinear relationship between remote sensing data and PMC. Linear regression models are highly susceptible to discrete values and cannot produce accurate predictions (Wei et al., 2025). During PLSR model training, improper selection of principal components may lead to overfitting of the training data, resulting in poor performance on test data (Shan et al., 2025).

Compared to RFR, SVR exhibits slightly lower estimation performance. While effective for certain problems, SVR is less suitable for handling large-scale datasets, is sensitive to parameter tuning, is computationally intensive, and demonstrates lower accuracy in the presence of high sample noise (Bai et al., 2025). Among the four machine learning algorithms evaluated, the RFR model achieved the highest coefficient of determination and the lowest prediction error. This performance can be attributed to the model’s ensemble of multiple decision trees, which effectively mitigates the influence of noise and outliers. However, the RFR model has certain limitations, including high computational demands and limited interpretability. Notably, the introduction of additional randomness during sample extraction and feature selection in the model construction process helps reduce the risk of overfitting, thereby enhancing model stability and estimation capability. Consequently, developing accurate and compact machine learning algorithms establishes a robust foundation for agricultural production and growth monitoring. This approach offers a simple and efficient method for quantitatively evaluating wheat PMC, demonstrating potential for broader application.

### 4.4 The effect of PMC on WUE

Globally, WUE is primarily associated with climatic factors such as precipitation conditions and air temperature. At the local scale, plant WUE is mainly related to the plant’s own biochemical characteristics and the availability of specific microclimate factors. The plant’s biochemical traits may influence its adaptability to the environment, while locally specific microclimate factors can also affect WUE. PMC directly influences growth and development. Both excessive and insufficient water may inhibit photosynthesis, root growth, and nutrient uptake, thereby reducing WUE. Healthy plants typically maintain optimal water content, sustaining high WUE. Compared to precipitation conditions, water availability exerts a greater influence on winter wheat WUE. Under high water content, plants can efficiently utilize soil moisture, maintain vigorous growth, and enhance WUE. However, as water content continues to increase, elevated stomatal conductance intensifies transpiration, while net photosynthetic capacity shows limited growth (Wang X. et al., 2023). This leads to reduced WUE, with the effect becoming more pronounced under high-moisture treatments. Conversely, in drought or waterlogged conditions, the water availability in the plant’s habitat decreases. To minimize water evaporation, reduce stomatal conductance, and improve WUE, other scholars both domestically and internationally have reached similar conclusions (Hao et al., 2025). Wang et al. (2017) found that water deficit significantly reduced plant water uptake and dry matter yield, resulting in comparable WUE but markedly lower nitrogen uptake compared to well-irrigated treatments. ET plays a crucial role in the water cycle and is the most significant factor influencing water content prediction, making a substantial positive contribution to its estimation. ET typically reflects plant health status, indicating robust growth under adequate moisture conditions. The integration of multi-source information, including VIs, TIs, and ET, provides robust data support for developing moisture content prediction models across different growth stages of winter wheat. This combination of physical characteristics offers more reliable foundations for the precise estimation of WUE, further enhancing model accuracy. It enables better capture of subtle variations in crop moisture dynamics, thereby improving the precision of WUE estimation at regional scales.

### 4.5 Uncertainty and future works

Although this study achieved promising results in PMC estimation through multi-source data fusion, several limitations remain that warrant further consideration. In terms of data acquisition and processing, while multiple features were extracted from UAV-mounted sensors, the exploration of thermal infrared information was not sufficiently comprehensive. Specifically, thermal infrared data were characterized using only a limited number of thermal indices, potentially overlooking critical information embedded in structural features, RGB color characteristics, and additional thermal indices—information that could further improve PMC estimation accuracy (Mao et al., 2023). Meanwhile, spectral and canopy thermal data often exhibit redundancy and overlap, which may interfere with the model’s ability to capture key discriminative information, thereby compromising the accuracy and reliability of final estimations and limiting the broader applicability of the findings (Sahoo et al., 2024). Despite these limitations, the PMC estimation model developed in this study demonstrated strong performance under high-throughput UAV platforms and holds considerable potential for extension to large-scale field breeding screening, precision irrigation decision support, and early detection of crop stress. Nevertheless, further validation of the model’s generalization capability across diverse ecological zones, crop varieties, and sensor configurations is essential. Future research should therefore focus on addressing the aforementioned data and modeling limitations and systematically evaluating model transferability under varying conditions to provide more robust data support and decision-making foundations for precision agriculture practices.

## 5 Conclusions

This study integrates multi-modal UAV remote sensing data, point cloud data, and LAI inversion models to construct an improved ET model. Utilizing four machine learning algorithms, it develops a PMC prediction model for winter wheat during critical growth stages. Combined with WUE, an irrigation scheduling optimization framework is established at the field scale. Key findings are as follows:

1. The correlations between VIs, ΔT, CWSI, and ET with PMC varied across the three critical growth stages of winter wheat, with ET exhibiting the highest correlations during the jointing and heading stages (|r| ≥ 0.639).
2. Compared to PMC prediction models constructed with different combinations of VIs, ET, VIs+ET, and VIs+TIs, the model employing the RFR algorithm with multimodal inputs (VIS+TIs+ET) demonstrated the best performance. Model accuracy progressively improved across growth stages, reaching its peak during grain filling (R^2^ = 0.900, nRMSE = 2.688%).
3. Under different water treatments, the highest WUE was achieved during the jointing stage under W3 treatment (PMC = 81.8%), during the heading stage under W1 treatment (PMC = 76.8%), and during the grain filling stage under W1 treatment (PMC = 64.0%).

In summary, this study employs an integrated approach combining UAV multimodal remote sensing with machine learning, alongside ET and TIs, to achieve high-precision monitoring of winter wheat moisture conditions. This provides new technical means for water management in precision agriculture, thereby significantly advancing the application and development of precision agriculture.

## Data Availability

The original contributions presented in the study are included in the article/supplementary material. Further inquiries can be directed to the corresponding author/s.
